# Discovery and fine-mapping of adiposity loci using high density imputation of genome-wide association studies in individuals of African ancestry: African Ancestry Anthropometry Genetics Consortium

**DOI:** 10.1371/journal.pgen.1006719

**Published:** 2017-04-21

**Authors:** Maggie C. Y. Ng, Mariaelisa Graff, Yingchang Lu, Anne E. Justice, Poorva Mudgal, Ching-Ti Liu, Kristin Young, Lisa R. Yanek, Mary F. Feitosa, Mary K. Wojczynski, Kristin Rand, Jennifer A. Brody, Brian E. Cade, Latchezar Dimitrov, Qing Duan, Xiuqing Guo, Leslie A. Lange, Michael A. Nalls, Hayrettin Okut, Salman M. Tajuddin, Bamidele O. Tayo, Sailaja Vedantam, Jonathan P. Bradfield, Guanjie Chen, Wei-Min Chen, Alessandra Chesi, Marguerite R. Irvin, Badri Padhukasahasram, Jennifer A. Smith, Wei Zheng, Matthew A. Allison, Christine B. Ambrosone, Elisa V. Bandera, Traci M. Bartz, Sonja I. Berndt, Leslie Bernstein, William J. Blot, Erwin P. Bottinger, John Carpten, Stephen J. Chanock, Yii-Der Ida Chen, David V. Conti, Richard S. Cooper, Myriam Fornage, Barry I. Freedman, Melissa Garcia, Phyllis J. Goodman, Yu-Han H. Hsu, Jennifer Hu, Chad D. Huff, Sue A. Ingles, Esther M. John, Rick Kittles, Eric Klein, Jin Li, Barbara McKnight, Uma Nayak, Barbara Nemesure, Adesola Ogunniyi, Andrew Olshan, Michael F. Press, Rebecca Rohde, Benjamin A. Rybicki, Babatunde Salako, Maureen Sanderson, Yaming Shao, David S. Siscovick, Janet L. Stanford, Victoria L. Stevens, Alex Stram, Sara S. Strom, Dhananjay Vaidya, John S. Witte, Jie Yao, Xiaofeng Zhu, Regina G. Ziegler, Alan B. Zonderman, Adebowale Adeyemo, Stefan Ambs, Mary Cushman, Jessica D. Faul, Hakon Hakonarson, Albert M. Levin, Katherine L. Nathanson, Erin B. Ware, David R. Weir, Wei Zhao, Degui Zhi, The Bone Mineral Density in Childhood Study (BMDCS) Group, Donna K. Arnett, Struan F. A. Grant, Sharon L. R. Kardia, Olufunmilayo I. Oloapde, D. C. Rao, Charles N. Rotimi, Michele M. Sale, L. Keoki Williams, Babette S. Zemel, Diane M. Becker, Ingrid B. Borecki, Michele K. Evans, Tamara B. Harris, Joel N. Hirschhorn, Yun Li, Sanjay R. Patel, Bruce M. Psaty, Jerome I. Rotter, James G. Wilson, Donald W. Bowden, L. Adrienne Cupples, Christopher A. Haiman, Ruth J. F. Loos, Kari E. North

**Affiliations:** 1 Center for Genomics and Personalized Medicine Research, Wake Forest School of Medicine, Winston-Salem, NC, United States of America; 2 Center for Diabetes Research, Wake Forest School of Medicine, Winston-Salem, NC, United States of America; 3 Department of Epidemiology, University of North Carolina, Chapel Hill, NC, United States of America; 4 The Charles Bronfman Institute for Personalized Medicine, Icachn School of Medicine at Mount Sinai, New York, NY, United States of America; 5 Department of Biostatistics, Boston University School of Public Health, Boston, MA, United States of America; 6 Department of Medicine, Johns Hopkins University School of Medicine, Baltimore, MD, United States of America; 7 Division of Statistical Genomics, Department of Genetics, Washington University School of Medicine, St. Louis MO, United States of America; 8 Department of Preventive Medicine, Keck School of Medicine, University of Southern California, Los Angeles, CA, United States of America; 9 Cardiovascular Health Research Unit, Department of Medicine, University of Washington, Seattle, WA, United States of America; 10 Division of Sleep and Circadian Disorders, Brigham and Women's Hospital, Boston, MA, United States of America; 11 Harvard Medical School, Boston, MA, United States of America; 12 Department of Genetics, University of North Carolina at Chapel Hill, Chapel Hill, NC, United States of America; 13 Institute for Translational Genomics and Population Sciences, Los Angeles Biomedical Research Institute at Harbor-UCLA Medical Center, Torrance, CA, United States of America; 14 Laboratory of Neurogenetics, National Institute on Aging, National Institutes of Health, Bethesda, MD, United States of America; 15 Data Tecnica International, Glen Echo, MD, United States of America; 16 National Institute on Aging, National Institutes of Health, Baltimore, MD, United States of America; 17 Department of Public Health Sciences, Stritch School of Medicine, Loyola University Chicago, Maywood, IL, United States of America; 18 Division of Endocrinology and Center for Basic and Translational Obesity Research, Boston Children's Hospital, Boston, MA, United States of America; 19 Broad Institute of MIT and Harvard, Cambridge, MA, United States of America; 20 Center for Applied Genomics, The Children’s Hospital of Philadelphia, Philadelphia, PA, United States of America; 21 Center for Research on Genomics and Global Health, National Human Genome Research Institute, National Institutes of Health, Bethesda, MD, United States of America; 22 Department of Public Health Sciences and Center for Public Health Genomics, University of Virginia School of Medicine, Charlottesville, VA, United States of America; 23 Division of Human Genetics, The Children’s Hospital of Philadelphia, Philadelphia, PA, United States of America; 24 Department of Epidemiology, University of Alabama at Birmingham, Birmingham, AL, United States of America; 25 Center for Health Policy and Health Services Research, Henry Ford Health System, Detroit, MI, United States of America; 26 Department of Epidemiology, University of Michigan, Ann Arbor, MI, United States of America; 27 Division of Epidemiology, Department of Medicine, Vanderbilt Epidemiology Center, Vanderbilt University School of Medicine, Nashville, TN, United States of America; 28 Division of Preventive Medicine, Department of Family Medicine and Public Health, University of California San Diego, La Jolla, CA, United States of America; 29 Department of Cancer Prevention and Control, Roswell Park Cancer Institute, Buffalo, NY, United States of America; 30 Department of Population Science, Rutgers Cancer Institute of New Jersey, New Brunswick, NJ, United States of America; 31 Cardiovascular Health Research Unit, Departments of Medicine and Biostatistics, University of Washington, Seattle, WA, United States of America; 32 Division of Cancer Epidemiology and Genetics, National Cancer Institute, Rockville, MD, United States of America; 33 Beckman Research Institute of the City of Hope, Duarte, CA, United States of America; 34 International Epidemiology Institute, Rockville, MD, United States of America; 35 Department of Translational Genomics, Keck School of Medicine, University of Southern California, Los Angeles, CA, United States of America; 36 Center for Human Genetics, University of Texas Health Science Center at Houston, Houston, TX, United States of America; 37 Department of Internal Medicine, Wake Forest School of Medicine, Winston-Salem, NC, United States of America; 38 SWOG Statistical Center, Fred Hutchinson Cancer Research Center, Seattle, WA, United States of America; 39 Program in Bioinformatics and Integrative Genomics, Harvard Medical School, Boston, MA, United States of America; 40 Sylvester Comprehensive Cancer Center, University of Miami Leonard Miller School of Medicine, Miami, FL, United States of America; 41 Department of Public Health Sciences, University of Miami Leonard Miller School of Medicine, Miami, FL, United States of America; 42 Department of Epidemiology, University of Texas M.D. Anderson Cancer Center, Houston, TX, United States of America; 43 Norris Comprehensive Cancer Center, University of Southern California, Los Angeles, CA, United States of America; 44 Cancer Prevention Institute of California, Fremont, CA, United States of America; 45 Department of Health Research and Policy (Epidemiology) and Stanford Cancer Institute, Stanford University School of Medicine, Stanford, CA, United States of America; 46 Division of Urology, Department of Surgery, The University of Arizona, Tucson, AZ, United States of America; 47 Glickman Urological and Kidney Institute, Cleveland Clinic, Cleveland, OH, United States of America; 48 Division of Cardiovascular Medicine, Department of Medicine, Stanford University School of Medicine, Palo Alto, CA, United States of America; 49 Cardiovascular Health Research Unit, Department of Biostatistics, University of Washington, Seattle, WA, United States of America; 50 Center for Public Health Genomics, University of Virginia School of Medicine, Charlottesville, VA, United States of America; 51 Department of Preventive Medicine, Stony Brook University, Stony Brook, NY, United States of America; 52 Department of Medicine, University of Ibadan, Ibadan, Nigeria; 53 Lineberger Comprehensive Cancer Center, University of North Carolina, Chapel Hill, Chapel Hill, NC, United States of America; 54 Department of Pathology and Norris Comprehensive Cancer Center, University of Southern California Keck School of Medicine, Los Angeles, CA, United States of America; 55 Department of Public Health Sciences, Henry Ford Health System, Detroit, MI, United States of America; 56 Department of Family and Community Medicine, Meharry Medical College, Nashville, TN, United States of America; 57 The New York Academy of Medicine, New York, NY, United States of America; 58 Division of Public Health Sciences, Fred Hutchinson Cancer Research Center, Seattle, WA, United States of America; 59 Department of Epidemiology, School of Public Health, University of Washington, Seattle, WA, United States of America; 60 Epidemiology Research Program, American Cancer Society, Atlanta, GA, United States of America; 61 Department of Epidemiology, Bloomberg School of Public Health, Baltimore, MD, United States of America; 62 Department of Epidemiology and Biostatistics, University of California, San Francisco, San Francisco, CA, United States of America; 63 Institute for Human Genetics, University of California, San Francisco, San Francisco, CA, United States of America; 64 Department of Epidemiology and Biostatistics, Case Western Reserve University, Cleveland, OH, United States of America; 65 Division of Cancer Epidemiology and Genetics, National Cancer Institute, National Institutes of Health, Bethesda, MD, United States of America; 66 Laboratory of Human Carcinogenesis, National Cancer Institute, Bethesda, MD, United States of America; 67 Department of Medicine, University of Vermont College of Medicine, Burlington, VT, United States of America; 68 Survey Research Center, Institute for Social Research, University of Michigan, Ann Arbor, MI, United States of America; 69 Department of Pediatrics, Perelman School of Medicine, University of Pennsylvania, Philadelphia, PA, United States of America; 70 Department of Medicine, University of Pennsylvania, Philadelphia, PA, United States of America; 71 School of Biomedical Informatics, University of Texas Health Science Center at Houston, Houston, TX, United States of America; 72 School of Public Health, University of Kentucky, Lexington, KY, United States of America; 73 Division of Endocrinology, The Children’s Hospital of Philadelphia, Philadelphia, PA, United States of America; 74 Center for Clinical Cancer Genetics, Department of Medicine and Human Genetics, University of Chicago, Chicago, IL, United States of America; 75 Division of Biostatistics, Washington University School of Medicine, St. Louis, MO, United States of America; 76 Department of Internal Medicine, Henry Ford Health System, Detroit, MI, United States of America; 77 Division of Gastroenterology, Hepatology and Nutrition, The Children’s Hospital of Philadelphia, Philadelphia, PA, United States of America; 78 Regeneron Genetics Center, Regeneron Pharmaceuticals, Inc, United States of America; 79 Departments of Genetics and Pediatrics, Harvard Medical School, Boston, MA, United States of America; 80 Department of Biostatistics, University of North Carolina at Chapel Hill, Chapel Hill, NC, United States of America; 81 Department of Computer Science, University of North Carolina at Chapel Hill, Chapel Hill, NC, United States of America; 82 Department of Medicine, University of Pittsburgh, Pittsburgh, PA, United States of America; 83 Cardiovascular Health Research Unit, Departments of Medicine, Epidemiology, and Health Services, University of Washington, Seattle, WA, United States of America; 84 Kaiser Permanente Washington Health Research Institute, Seattle, WA, United States of America; 85 Division of Genomic Outcomes, Departments of Pediatrics and Medicine, Los Angeles Biomedical Research Institute at Harbor-UCLA Medical Center, Los Angeles, CA, United States of America; 86 Department of Physiology and Biophysics, University of Mississippi Medical Center, Jackson, MS, United States of America; 87 Department of Biochemistry, Wake Forest School of Medicine, Winston-Salem, NC, United States of America; 88 NHLBI Framingham Heart Study, Framingham, MA, United States of America; 89 The Mindich Child Health and Development Institute, Ichan School of Medicine at Mount Sinai, New York, NY, United States of America; The University of North Carolina at Chapel Hill, UNITED STATES

## Abstract

Genome-wide association studies (GWAS) have identified >300 loci associated with measures of adiposity including body mass index (BMI) and waist-to-hip ratio (adjusted for BMI, WHR_adjBMI_), but few have been identified through screening of the African ancestry genomes. We performed large scale meta-analyses and replications in up to 52,895 individuals for BMI and up to 23,095 individuals for WHR_adjBMI_ from the African Ancestry Anthropometry Genetics Consortium (AAAGC) using 1000 Genomes phase 1 imputed GWAS to improve coverage of both common and low frequency variants in the low linkage disequilibrium African ancestry genomes. In the sex-combined analyses, we identified one novel locus (*TCF7L2/HABP2*) for WHR_adjBMI_ and eight previously established loci at *P* < 5×10^−8^: seven for BMI, and one for WHR_adjBMI_ in African ancestry individuals. An additional novel locus (*SPRYD7/DLEU2*) was identified for WHR_adjBMI_ when combined with European GWAS. In the sex-stratified analyses, we identified three novel loci for BMI (*INTS10/LPL* and *MLC1* in men, *IRX4/IRX2* in women) and four for WHR_adjBMI_ (*SSX2IP*, *CASC8*, *PDE3B* and *ZDHHC1/HSD11B2* in women) in individuals of African ancestry or both African and European ancestry. For four of the novel variants, the minor allele frequency was low (<5%). In the trans-ethnic fine mapping of 47 BMI loci and 27 WHR_adjBMI_ loci that were locus-wide significant (*P* < 0.05 adjusted for effective number of variants per locus) from the African ancestry sex-combined and sex-stratified analyses, 26 BMI loci and 17 WHR_adjBMI_ loci contained ≤ 20 variants in the credible sets that jointly account for 99% posterior probability of driving the associations. The lead variants in 13 of these loci had a high probability of being causal. As compared to our previous HapMap imputed GWAS for BMI and WHR_adjBMI_ including up to 71,412 and 27,350 African ancestry individuals, respectively, our results suggest that 1000 Genomes imputation showed modest improvement in identifying GWAS loci including low frequency variants. Trans-ethnic meta-analyses further improved fine mapping of putative causal variants in loci shared between the African and European ancestry populations.

## Author summary

Genome-wide association studies (GWAS) have identified >300 genetic regions that influence body size and shape as measured by body mass index (BMI) and waist-to-hip ratio (WHR), respectively, but few have been identified in populations of African ancestry. We conducted large scale high coverage GWAS and replication of these traits in 52,895 and 23,095 individuals of African ancestry, respectively, followed by additional replication in European populations. We identified 10 genome-wide significant loci in all individuals, and an additional seven loci by analyzing men and women separately. We combined African and European ancestry GWAS and were able to narrow down 43 out of 74 African ancestry associated genetic regions to contain small number of putative causal variants. Our results highlight the improvement of applying high density genome coverage and combining multiple ancestries in the identification and refinement of location of genetic regions associated with adiposity traits.

## Introduction

Obesity is a worldwide public health epidemic, with current US estimates of 37.9% obese and 7.7% morbidly obese adults [1]. Disparities in obesity rates, as well as rates of comorbidities and mortality, are evident across sex and racial/ethnic groups. Estimates from NHANES for 2013–2014 [1] show that obesity is more prevalent among African Americans (48.5%) than among non-Hispanic Whites (37.1%). In addition, obesity rates are higher among African American women (57.2%) than among African American men (38.2%). For comparison, the obesity rates in non-Hispanic Whites were 38.7% and 35.4%, respectively, for women and men.

Genome-wide association studies (GWAS) in diverse populations have identified > 300 loci associated with measures of adiposity including body mass index (BMI) and waist-to-hip ratio (adjusted for BMI, WHR_adjBMI_) in populations of European [2–9], African [10–12], and East Asian ancestry [13–15]. The majority of associated variants are common (MAF >5%) with small effect size, and jointly explain only a fraction of the phenotypic variances [7–8]. It has long been hypothesized that low frequency (MAF = 0.5–5%) and rare (MAF < 0.5%) variants may also contribute to variability in complex traits. However, these variants are not well captured in previous GWAS imputed to the HapMap reference panel [16–17]. The availability of higher density reference panels such as the 1000 Genomes Project (38M variants in 1092 individuals from phase 1) [18] has demonstrated improved imputation quality in European populations particularly for low frequency variants (aggregate R^2^ ~0.6 for MAF = 0.5%). However its impact is less clear for non-European populations [19]. We took this opportunity to use higher density imputation to reevaluate our previous GWAS for associations with anthropometric traits in individuals of African ancestry (AA) including African Americans and Africans.

The African Ancestry Anthropometry Genetics Consortium (AAAGC) previously identified seven genome-wide significant loci for BMI in up to 71,412 AA individuals, and an additional locus when combined with European ancestry (EA) data from the Genetic Investigation of ANthropometric Traits (GIANT) consortium using GWAS imputed to the HapMap Phase 2 reference panel [11]. No genome-wide significant loci were identified for WHR_adjBMI_ in a GWAS of up to 27,350 AA individuals [12]. The low yield of discovery in AA studies is likely due to their relatively smaller sample sizes in comparison to EA studies [7–8], as well as their lower degree of linkage disequilibrium (LD) and thus poorer imputation quality. Here, we extended our previous work in the AAAGC to perform meta-analyses and replication of GWAS imputed to the 1000 Genomes reference panel in up to 52,895 AA individuals for BMI and up to 23,095 AA individuals for WHR_adjBMI_. We aimed to 1) discover novel variants, 2) fine map established loci, and 3) evaluate the coverage and contribution of low frequency variants in genetic associations in AA populations.

## Results

### Study overview

We conducted sex-combined and sex-stratified meta-analyses of GWAS summary statistics across 17 studies for BMI (N = 42,752) and 10 studies for WHR_adjBMI_ (N = 20,384) in AA individuals in stage 1 discovery (S1 and S2 Tables, S1 Fig). Missing genotypes in individual studies were imputed to the 1000 Genomes Project cosmopolitan reference panel (Phase I Integrated Release Version 3, March 2012) [18] using MaCH/minimac [20] or SHAPEIT2/IMPUTEv2 [21–22] (S3 Table). Among all variants with MAF ≥ 0.1% in the largest Women’s Health Initiative (WHI) study, the average info score was 0.81 and 90.5% had imputation info score ≥ 0.3 (S4 Table). Genomic control corrections were applied to each study and after meta-analysis (λ = 1.07 for BMI, 1.01 for WHR_adjBMI_) (S3 Table, S2–S5 Figs). Association results for ~18M variants for BMI and ~21M variants for WHR_adjBMI_ were subsequently interrogated further.

From stage 1 meta-analyses, variants associated with BMI (3,241 in all, 1,498 in men, 2,922 in women) and WHR_adjBMI_ (2,496 in all, 1,408 in men, 2,827 in women) at *P* < 1×10^−4^ were carried forward for replication in AA and EA. Stage 2 included 10,143 AA (2,458 men and 7,685 women) for BMI and 2,711 AA (981 men and 1,730 women) for WHR_adjBMI_ analyses. Stage 3 included 322,154 EA (152,893 men and 171,977 women) for BMI and 210,086 EA (104,079 men and 116,742 women) for WHR_adjBMI_ analyses by imputing HapMap summary statistics results [7–8] to 1000 Genomes [23] (S1 Fig). Meta-analyses were performed to combine either sex-combined or sex-specific results from AA (stages 1+2, N ≤ 57,895 for BMI, ≤ 23,095 for WHR_adjBMI_ in sex-combined analyses) and both AA and EA (stages 1+2+3, N ≤ 380,049 for BMI, ≤ 233,181 for WHR_adjBMI_ in sex-combined analyses, S6–S9 Figs). Variants that reached genome-wide statistical significance (*P* < 5×10^−8^) were assessed for generalization of associations with BMI to children in two additional AA cohorts (N = 7,222).

### Genome-wide significant loci in meta-analyses

#### Sex-combined analyses

**Fig 1:**
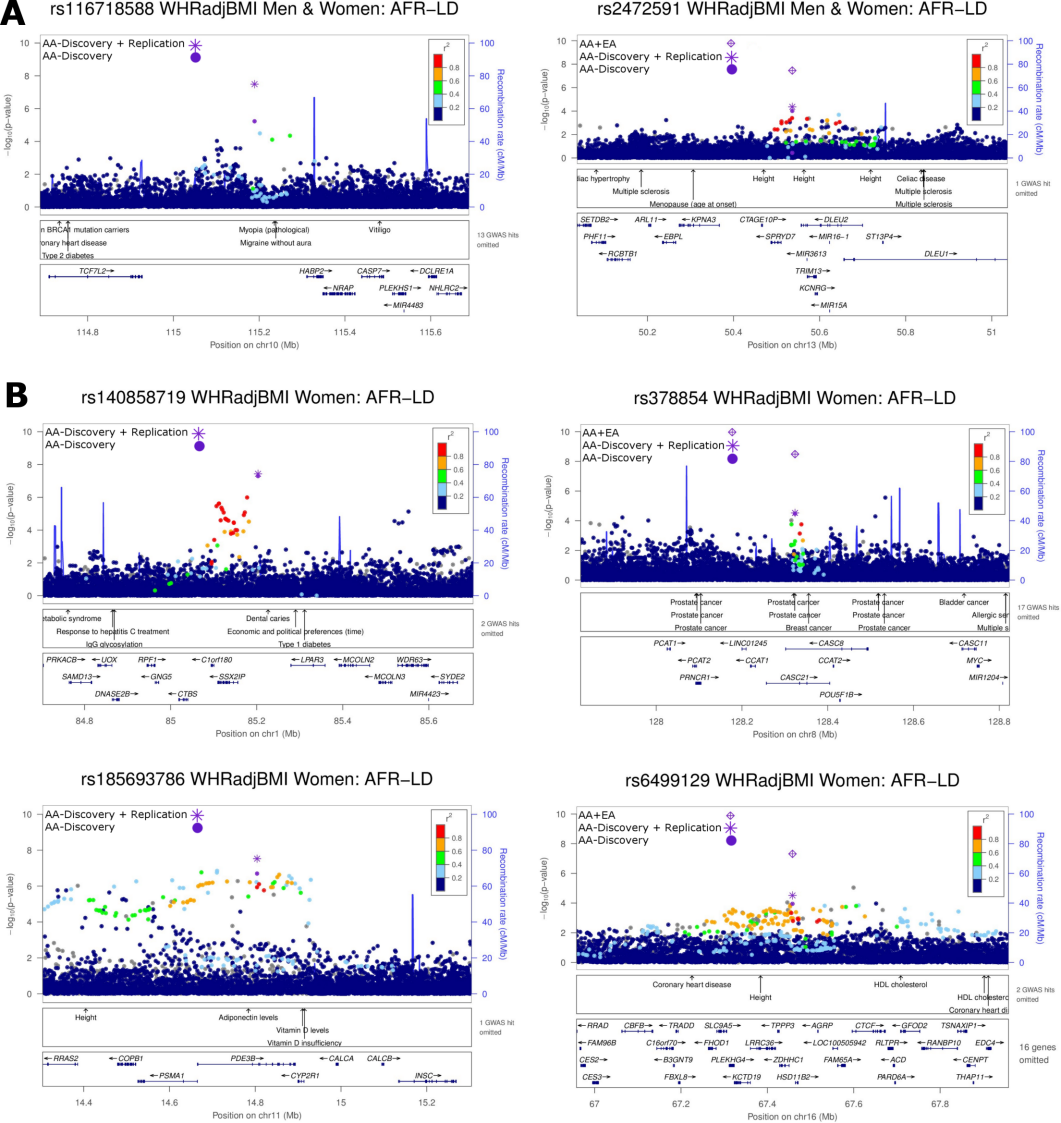
Locuszoom plots of six novel waist-to-hip ratio adjusted for BMI (WHR_adjBMI_) loci: (A) *TCF7L2/HABP2* and *SPRYD7/DLEU2* in men and women combined; and (B) *SSX2IP*, *PDE3B*, *CASC8*, and *ZDHHC1/HSD11B2* in women only. All plots use AFR LD from the 1000 Genomes phase 1 reference panel. In each plot, the most significant variant within a 1Mb regional locus is highlighted. *P*-values for all variants including the most significant variant are based on the African ancestry discovery phase only (AA-Discovery). In addition, for the most significant variant, *P*-values are annotated and illustrated from the African ancestry discovery and replication phases (AA-Discovery+Replication). SNP rs2472591 was available in the Europeans from the GIANT consortium effort and combined with the African ancestry discovery and replication phases (AA+EA).

In the sex-combined meta-analysis of BMI in AA, seven previously established European or African ancestry-derived loci in/near *SEC16B*, *TMEM18*, *GNPDA2*, *GALNT10*, *KLHL32*, *FTO* and *MC4R* reached genome-wide significance (*P* < 5×10^−8^) (Table 1, S6 and S10A Figs). The rs7708584 variant at *GALNT10* had the lowest *P*-value (*P* = 4.2×10^−14^) and was the same lead variant as reported in our previous AA study (S5 Table) [11]. The association at *KLHL32* was specific to the AA population as the lead variant was not statistically significant in EA (*P* > 0.05), consistent with our previous finding (S5 Table) [11]. No additional novel BMI loci were identified after meta-analysis of AA and EA data. Two previously reported loci in AA, *ADCY3* and *MIR148A-NFE2L3* [11], did not reach genome-wide significance in the present study. The previously reported lead variant at *ADCY3*, rs7586879, showed weaker effect and association in AA (effect = 0.047, *P* = 3.60×10^−8^ [11] vs. effect = 0.032, *P* = 1.05×10^−4^, this study). On the other hand, a moderately correlated (r^2^ = 0.52 in AFR) variant, rs10203482, show stronger association at *P* = 3.35×10^−7^ (S5 Table). At *MIR148A/NFE2L3*, the previously reported lead variant identified by meta-analysis of AA and EA, rs10261878, also showed weaker association in the current study primarily due to weak association in EA (*P* = 4.10×10^−5^ in AA, 4.69×10^−3^ in EA and 2.10×10^−5^ in AA+EA) (S5 Table). For WHR_adjBMI_, an established locus (*ADAMTS9-AS2*) (S10B Fig) and a novel locus (*TCF7L2/HABP2*) (Fig 1A) showed significant associations, the latter lead variant rs116718588 was low frequency (MAF = 0.045, Table 1). Meta-analyses including both AA and EA individuals revealed an additional novel locus at *SPRYD7/DLEU2* for WHR_adjBMI_ (Table 1, Fig 1A and S7 Fig). Overall, all the BMI associated lead variants were present in HapMap and were in high LD (r^2^ > 0.5 in 1000 Genomes AFR population) with the lead variants in our previous HapMap imputed data, except for *TMEM18* rs62105306 (r^2^ = 0.17) which is absent in HapMap. In contrast, all three WHR_adjBMI_ lead variants were absent in HapMap and the lead variants at *ADAMTS9-AS2* and *TCF7L2-HABP2* were in low LD (r^2^ < 0.5) with the lead variants in our previous HapMap imputed data [11–12]. We used conditional and joint association analyses to examine the genome-wide significant locus for secondary signals, but no additional independent signals were found.

**Table 1:** Novel and previously identified BMI and WHR_adjBMI_ loci at *P* < 5×10^−8^ in African ancestry discovery and replication samples, and European ancestry replication samples.

Lead variant by locus									AA Discovery				AA Replication				AA Discovery + Replication		EA		AA + EA
Trait	Lead SNP	Chr	Position (b37/hg19)	Known Locus (if Yes, lead published variant)		Locus	Effect/Other alleles	EAF	Effect (SE)	*P*	HetISq	N	Effect (SE)	*P*	HetISq	N	Effect (SE)	*P*	*P*	N	*P*
BMI	rs543874	1	177,889,480	Yes	rs543874	*SEC16B*	G/A	0.248	0.055 (0.008)	5.75E-11	0	42,681	0.057 (0.017)	6.59E-04	28.9	10,143	0.055 (0.008)	1.76E-13	4.36E-35	322,008	6.35E-46
BMI	rs62105306	2	633,660	Yes	rs13021737	*TMEM18*	T/C	0.751	0.056 (0.01)	1.55E-08	0	41,492	0.04 (0.02)	4.40E-02	49.4	10,143	0.053 (0.009)	2.17E-09	6.10E-21	244,176	3.04E-28
BMI	rs10938397	4	45,182,527	Yes	rs10938397	*GNPDA2*	G/A	0.243	0.053 (0.008)	3.76E-10	3.6	42,752	0.011 (0.017)	5.40E-01	54	10,143	0.044 (0.008)	3.95E-09	1.87E-38	320,955	5.60E-46
BMI	rs7708584	5	153,543,466	Yes	rs7715256; rs7708584 ^a^	*GALNT10*	A/G	0.307	0.059 (0.008)	1.05E-13	4.6	42,750	0.034 (0.016)	3.93E-02	6.2	10,143	0.054 (0.007)	4.21E-14	3.80E-07	234,015	4.35E-15
BMI	rs17057164	6	97,410,536	Yes	rs974417 ^a^	*KLHL32*	T/C	0.659	0.043 (0.008)	1.75E-08	0	42,751	0.025 (0.015)	9.97E-02	35.2	10,143	0.04 (0.007)	6.08E-09	7.44E-01	233,997	5.43E-03
BMI	rs17817964	16	53,828,066	Yes	rs1558902	*FTO*	T/C	0.117	0.067 (0.011)	5.48E-09	0	42,750	0.08 (0.025)	1.19E-03	49.2	10,143	0.069 (0.01)	2.72E-11	2.40E-139	321,602	1.13E-146
BMI	rs6567160	18	57,829,135	Yes	rs6567160	*MC4R*	C/T	0.197	0.062 (0.009)	2.74E-11	35.3	42,750	0.044 (0.019)	1.99E-02	33.5	10,143	0.059 (0.008)	2.29E-12	8.23E-54	321,958	2.09E-64
WHR_adjBMI_	rs66815886	3	64,703,394	Yes	rs2371767	*ADAMTS9-AS2*	G/T	0.457	0.07 (0.01)	3.90E-12	0	20,383	0.005 (0.033)	8.75E-01	42.1	2,711	0.064 (0.01)	2.46E-11	5.17E-19	145,257	9.13E-27
WHR_adjBMI_	rs116718588	10	115,189,239	No		*TCF7L2/HABP2*	A/G	0.955	0.114 (0.025)	5.88E-06	0	20,384	0.348 (0.084)	3.82E-05	45.6	2,711	0.134 (0.024)	3.22E-08	NA	NA	NA
WHR_adjBMI_	rs2472591	13	50,536,360	No		*SPRYD7/DLEU2*	T/A	0.206	0.05 (0.013)	9.72E-05	0	20,371	0.06 (0.049)	2.21E-01	0	2,160	0.05 (0.012)	4.36E-05	1.69E-05	140,431	3.53E-08

AA: African ancestry; BMI: body mass index; Chr: chromosome; EA: European ancestry; EAF: effect allele frequency; HetISq: heterogeneity measured by I-square; SE: standard error; WHR_adjBMI_: waist-to-hip ratio adjusted for BMI

^a^ lead published variants reported in African ancestry

#### Sex-stratified analyses

**Fig 2:**
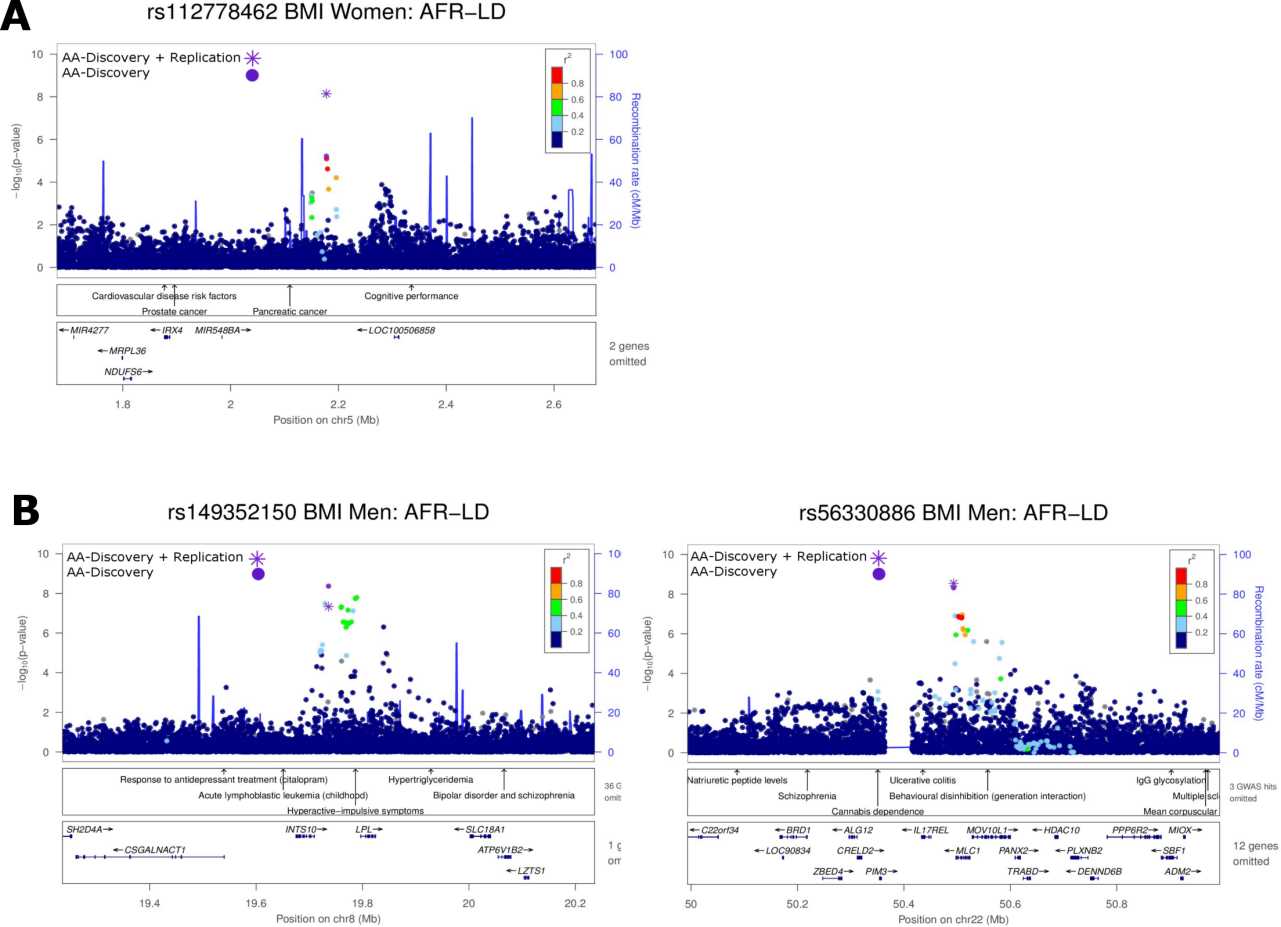
Locuszoom plots for three novel BMI loci: (A) *IRX4/IRX2* in women only; and (B) *INTS10/LPL* and *MLC1* in men only. All plots use AFR LD from the 1000 Genomes phase 1 reference panel. In each plot, the most significant variant within a 1Mb regional locus is highlighted. *P*-values for all variants including the most significant variant are based on the African ancestry discovery phase only (AA-Discovery). In addition, for the most significant variant, *P*-values are annotated and illustrated from the African ancestry discovery and replication phases (AA-Discovery+Replication).

In the sex-stratified meta-analysis in AA, four established BMI loci (*SEC16B*, *GALNT10*, *FTO* and *MC4R*) and one established WHR_adjBMI_ locus (*ADAMTS9-AS2*) were genome-wide significant among women (S6 Table, S8 and S9 Figs). *ADAMTS9-AS2* showed a stronger association with WHR_adjBMI_ among women than among men (*P*_*het*_ = 0.02) (S6 Table), consistent with findings among EA [9]. On the other hand, although our observed *SEC16B* rs543874 effect size differences (0.064 vs. 0.038, *P*_*het*_ = 0.08) for BMI in women compared to men were similar to those previously observed among EA (0.060 vs. 0.034, *P*_*het*_ = 5.23×10^−5^) [7], we did not observe statistically significant differences in effect size, likely due to a much smaller sample size and thus lower statistical power in our study. All these five loci were also genome-wide significant in the sex-combined meta-analyses. They were not further examined in subsequent sex-stratified analyses given their smaller sample sizes compared to the sex-combined analyses. In AA, additional novel loci were observed for association with BMI; these were variants in *IRX4/IRX2* among women, variants in *INTS10/LPL* and *MLC1* among men (Fig 2), and for WHR_adjBMI_, variants in *SSX2IP* and *PDE3B* among women (Fig 1B, Table 2). In meta-analyses including both AA and EA, two additional novel loci at *CASC8* and *ZDHHC1/HSD11B2* were identified for WHR_adjBMI_ in women (Table 2, Fig 1B). Among all loci, the effect sizes of six variants (*IRX4/IRX2*, *INTS10/LPL*, *MLC1*, *ADAMTS9-AS2*, *PDE3B* and *CASC8*) were nominally significant different between men and women in AA (*P*_*het*_ < 0.05) (S6 Table).

**Table 2:** Additional novel BMI and WHR_adjBMI_ loci at *P* < 5×10^−8^ in sex-stratified analyses of African ancestry discovery and replication samples.

Lead variant by locus								AA Discovery				AA Replication				AA Discovery + Replication			EA		AA + EA
Trait	Cohort	Lead SNP	Chr	Position (b37/hg19)	Locus	Effect/Other alleles	EAF	Effect (SE)	*P*	HetISq	N	Effect (SE)	*P*	HetISq	N	Effect (SE)	*P*	N	*P*	N	*P*
BMI	Women	rs112778462	5	2,177,693	*IRX4/ IRX2*	A/G	0.023	0.159 (0.035)	6.06E-06	35.4	25,792	0.219 (0.059)	2.11E-04	47.6	6,984	0.175 (0.03)	7.21E-09	32,776	NA	NA	NA
BMI	Men	rs149352150	8	19,736,154	*INTS10/ LPL*	G/A	0.013	0.341 (0.058)	4.29E-09	0	15,179	-0.034 (0.163)	8.35E-01	1.0	2,147	0.299 (0.055)	4.68E-08	17,326	NA	NA	NA
BMI	Men	rs56330886	22	50,493,427	*MLC1*	G/T	0.189	0.096 (0.016)	4.81E-09	3.3	15,721	0.063 (0.052)	2.21E-01	0.2	2,147	0.093 (0.016)	2.88E-09	17,868	NA	NA	NA
WHR_adjBMI_	Women	rs140858719	1	85,203,061	*SSX2IP*	G/A	0.994	0.506 (0.093)	5.07E-08	0	11314	0.403 (0.487)	4.08E-01	0	834	0.502 (0.091)	3.69E-08	12,148	NA	NA	NA
WHR_adjBMI_	Women	rs378854	8	128,323,819	*CASC8*	C/T	0.787	0.062 (0.015)	3.34E-05	4.5	15,600	0.034 (0.054)	5.29E-01	73.2	1,730	0.060 (0.014)	2.99E-05	17,330	3.70E-06	85325	3.26E-09
WHR_adjBMI_	Women	rs185693786	11	14,804,296	*PDE3B*	G/A	0.930	0.122 (0.023)	2.01E-07	0	15,601	0.17 (0.085)	4.50E-02	21.6	1,730	0.125 (0.023)	2.98E-08	17,331	NA	NA	NA
WHR_adjBMI_	Women	rs6499129	16	67,458,251	*ZDHHC1/ HSD11B2*	A/C	0.434	0.045 (0.012)	1.12E-04	30.9	15,588	0.071 (0.042)	9.29E-02	41.2	1,730	0.047 (0.011)	3.07E-5	17,318	4.13E-05	86328	4.84E-08

AA: African ancestry; BMI: body mass index; Chr: chromosome; EA: European ancestry; EAF:effect allele frequency; HetISq: heterogeneity measured by I-square; SE: standard error; WHR_adjBMI_: waist-to-hip ratio adjusted for BMI

#### Replication in children

We evaluated the seven sex-combined and three sex-specific genome-wide significant BMI loci for associations in 7,222 AA children (3,552 boys and 3,670 girls). All lead variants displayed directional consistency, and five of these including *SEC16B*, *TMEM18*, *GNPDA2*, *GALNT10* and *MC4R* showed nominal associations with BMI (*P* < 0.05, *P*_*binomial*_ = 4.70×10^−8^) (S7 Table), supporting the role of these loci in modulating adiposity in AA children. WHR_adjBMI_ data were not available in the cohorts of children.

#### Functional characterization of novel loci

We used multiple complementary approaches to elucidate the putative causal genes and/or variants associated with the nine novel BMI and WHR_adjBMI_ loci from the sex-combined and sex-stratified analyses, including annotating nearby coding variants, cis-expression quantitative trait loci (cis-eQTL) analyses, and functional regulatory genomic element analyses. One missense variant in *PLEKHG4*, rs8044843, was in high LD (r^2^ = 0.75 in AFR) with rs6499129 associated with WHR_adjBMI_ in women (S8 Table). We did not identify any coding variants in high LD (r^2^>0.7) with other lead variants within the flanking 1Mb-regions. Regulatory element analyses using RegulomeDB [24] and HaploReg [25] revealed that proxies (r^2^ = 0.73–0.84) to lead variants at three WHR_adjBMI_ loci (*SPRYD7/DLEU2*, *PDE3B*, and *ZDHHC1/HSD11B2)* were associated with transcription factor binding, DNase peak, promoter or enhancer histone marks (S8 Table). In addition, the lead variant rs2472591 at *SPRYD7/DLEU2* was in high LD (r^2^ = 0.85) with rs790943, a cis-eQTL associated with expression of the nearby gene, *TRIM13*, in blood dendritic cells in tuberculosis patients [26] (S9 Table), suggesting the associations at the *SPRYD7/DLEU2* locus may be involved in the regulation of nearby gene expression at *TRIM13*.

#### Cross-trait associations of novel loci

We searched the NHGRI-EBI GWAS [27] and Genome-Wide Repository of Associations Between SNPs and Phenotypes (GRASP) [28] catalogs to assess if any of the nine novel lead variants were in high LD with variants that were genome-wide significantly (*P* < 5×10^−8^) or nominally (*P* < 0.05) associated with related anthropometric and cardiometabolic traits or gene expression in prior studies. Although a few lead variants were physically close (<500 kb) to GWAS loci for related traits in the NHGRI-EBI GWAS Catalog (Figs 1 and 2), none of our lead variants were in high LD with the previously associated lead variants. Additionally, there were no nearby associations for novel BMI loci in the GRASP Catalog. Of the novel variants associated with WHR_adjBMI_, rs2472591 at *SPRYD7/DLEU2*, rs378854 near *MYC*, and rs6499129 near *ZDHHC1/HSD11B2* were in high LD (r^2^ > 0.7) with previously-reported WHR_adjBMI_ variants, but they did not reach genome-wide significance (*P* > 2×10^−5^) [3] (S9 Table). Other nearby associations with related cardiometabolic traits include chronic kidney disease (CKD), high density lipoprotein cholesterol (HDL-C), anthropometric traits (BMI, height, and birth weight), blood pressure (systolic blood pressure and hypertension), diabetes-related traits (blood glucose and HOMA-IR), and gene expression of several genes (e.g. *ATP6V0D1*, *ZDHHC1*, *DUS2L*, *AGRP*, *GFOD2* and *LRRC29*).

### Evaluation of established European loci in African ancestry populations

#### Conditional analysis in GWAS loci

Among the six BMI (*SEC16B*, *TMEM18*, *GNPDA2*, *GALNT10*, *FTO* and *MC4R*) and one WHR_adjBMI_ (*ADAMTS9-AS2*) genome-wide significant loci in AA that were previously reported in EA [7–8], we tested whether the African derived lead variants were independent of the reported European signals by conditioning on the European lead variants or their surrogates. For three of the BMI loci (*SEC16B*, *GNPDA2* and *MC4R*), our lead variants are the same as those reported in the previous literature [7]. For all other loci, the lead variants demonstrated substantially lower significance upon conditional analysis, suggesting that the African ancestry results represented the same association signals as previously reported in GWAS performed predominantly in EA populations (S10 Table).

#### SNP transferability

We further examined all sex-combined and sex-stratified BMI and WHR_adjBMI_ loci identified from previous EA studies [7–9] in our AA data. Among 176 EA lead variants from 170 BMI loci, 119 variants displayed directionally consistent associations with BMI in our data, 31 of these were nominally significant at *P* < 0.05 (*P*_*binomial*_ = 2.2×10^−18^ among 176 variants). Among 84 EA lead variants from 65 WHR_adjBMI_ loci, 69 variants displayed directionally consistent associations with WHR_adjBMI_, and 23 of these were nominally significant (*P*_*binomial*_ = 5.3×10^−19^ among 84 variants) (S11 Table). EA lead variants in 11 BMI and 3 WHR_adjBMI_ loci showed directional consistency and significant associations after correction for multiple comparisons (*P* < 1.92×10^−4^). Among the 54 nominally transferable lead variants for BMI and WHR_adjBMI_, 45% and 43% of the effect sizes, respectively, were larger in the EA than the AA populations. In addition, 65% of the frequencies of the trait-raising alleles were higher in the EA populations for both traits. The correlations of both effect sizes and allele frequency of the transferable variants were high (0.74 and 0.79, respectively) for BMI but weak (0.19 and 0.37, respectively) for WHR_adjBMI_ (S11 Fig). The significant but low proportion of lead variants that were transferable from EA to AA (18% for BMI and 27% for WHR_adjBMI_) suggests either that many loci are not implicated in AA or population differences in LD mask the detection of associated variants in AA. On the other hand, those variants that were transferable explain similar levels of variances for BMI in both populations, but not for WHR_adjBMI_.

#### Locus transferability

We further investigated locus transferability in EA loci derived from sex-combined and sex-stratified analyses by considering varying LD between EA and AA populations. S12 Table reports the most significant lead regional variants in our AA sex-combined and sex-stratified data within 0.1cM region of the previously published EA loci (from 176 BMI and 84 WHR_adjBMI_ lead variants) [7–8]. Forty-five (26%) lead regional variants from BMI loci remained significant (*P*_*locus*_ < 0.05) after adjustment for the number of independent variants tested at each locus. Sixteen (36%) and 22 (49%) of these 45 lead regional variants are in LD (r^2^>0.2) with the EA BMI lead variants using 1000 Genomes AFR and CEU LD, respectively. Twenty-five of these variants are highly correlated with EA lead variants(r^2^>0.7 in CEU) or had ≥1 standard error decrease in effect sizes after conditional analyses, representing same association signals as in EA populations. Twenty-one (32%) lead regional variants for WHR_adjBMI_ loci remained significant. Nine (43%) and seven (33%) of the 21 lead regional variants was in LD with the EA lead variant using 1000 Genomes AFR and CEU LD, respectively. Seven of these variants represented the same EA association in conditional analyses (S12 Table).

### Fine mapping of novel AA loci and EA-AA transferable established loci

Among the locus-wide significant established loci (44 for BMI given two of 45 lead regional variants were identical in two loci, and 21 for WHR_adjBMI_), and novel loci (three for BMI and six for WHR_adjBMI_) derived from the sex-combined and sex-stratified analyses, we performed fine mapping to localize putative causal variants. We constructed 99% credible sets containing variants that jointly account for 99% posterior probability of driving the association in a locus using the corresponding sex-combined or sex-stratified meta-analysis results from AA, EA and combined ancestry (S13 Table). A smaller number of variants in a credible set represent a higher resolution of fine mapping and we considered a credible set containing ≤ 20 variants as “tractable’ for follow up. The credible sets in the EA analyses were generally smaller than those in the AA given their larger sample size. As compared to the EA analyses, the number of tractable loci in the meta-analyses of AA and EA increased from 23 to 26 for BMI, and from 14 to 17 for WHR_adjBMI_.

Among these 43 tractable loci, the lead variants in the combined ancestry analyses had posterior probability ≥ 0.95 in six BMI loci (*SEC16B*, *TLR4*, *STXBP6*, *NLRC3*, *FTO* and *MC4R*) and seven WHR_adjBMI_ loci (*DCST2*, *PPARG*, *ADAMTS9*, *SNX10*, *KLF13*, *CMIP* and *PEMT*) (S13 Table). Functional characterization of variants within the tractable credible sets revealed two loci contain nonsynonymous variants (*ADCY3*: rs11676272 S107P; *SH2B1*: rs7498665 T484A from the *ATP2A1* locus), but they had low posterior probability to drive the respective associations (0.02 and 0.15, respectively) (S14 Table). On the other hand, the *ADCY3* non-coding variants rs10182181 and rs6752378 had higher posterior probability (0.26–0.72) and are cis-eQTLs of *ADCY3* and nearby genes. Several BMI loci including *MTCH2*, *MAP2K5*, *NLRC3* and *ATP2A1*, and WHR_adjBMI_ loci including *TBX15-WARS2* and *FAM13A*, also contained cis-eQTL variants regulating nearby gene expression in subcutaneous and/or visceral adipose tissue (S14 Table).

## Discussion

In our large-scale meta-analyses of GWAS in up to 52,895 and 23,095 individuals of African ancestry for BMI and WHR_adjBMI_, respectively, we identified three novel (*IRX4/IRX2*, *INTS10/LPL* and *MLC1*) and seven established (*SEC16B*, *TMEM18*, *GNPDA2*, *GALNT10*, *KLHL32*, *FTO* and *MC4R*) BMI loci, as well as three novel (*TCF7L2/HABP2*, *SSX2IP* and *PDE3B*) and one established (*ADAMTS9-AS2*) WHR_adjBMI_ loci in either sex-combined or sex-stratified analyses. By employing a recently developed method [23] to impute European GWAS summary statistics to the denser 1000 Genomes reference panel, followed by meta-analyses of both African and European ancestry individuals, we also identified three additional novel loci (*SPRYD7/DLEU2*, *CASC8* and *ZDHHC1/ HSD11B2*) for WHR_adjBMI_. While all lead variants from established loci are common (MAF ≥ 5%), four of the nine lead variants from novel loci were low frequency (0.5% ≤ MAF < 5%). In addition, the lead variants from established loci including *TMEM18* and *ADAMTS9-AS2* were absent in HapMap. Overall, these results suggest the deeper genome coverage and/or improved imputation quality using 1000 Genomes, and complemented with additional sex-stratified analyses, facilitate the discovery of novel loci and identification of variants with stronger effects in established loci.

Among the novel sex-specific BMI loci (*IRX4/IRX2*, *INTS10/LPL* and *MLC1*), we did not identify any putative coding variants or regulatory regions underlying our association signals. Additionally, no associations have been reported with other metabolic traits in these novel BMI-associated signals. The first lead variant rs112778462 is located between the *IRX4* and *IRX2* genes which are members of the Iroquois homeobox gene family. *IRX2* expression has been associated with deposition of fat in the subcutaneous abdominal adipose tissue but no sex difference was observed [29–30]. *Irx4* knock out mice demonstrated cardiomyopathy with compensated increased *Irx2* expression [31]. The second lead variant rs149352150 is located between the *INTS10* and *LPL* genes. *LPL* encoded lipoprotein lipase is expressed in several tissues including adipose to mediate triglyceride hydrolysis and lipoprotein uptake. The serum LPL mass [32] and LPL activity and fat cell size of adipose tissues at gluteus and thigh [33] have been reported to be higher in women than in men. Previous GWAS demonstrated association of *LPL* with triglycerides and HDL cholesterol [34–35]. However, the reported lead variant rs12678919 was not in strong LD with rs149352150 (r^2^ = 0.005 in AFR and 0.006 in EUR). The third lead variant rs56330886 is located in a gene-rich region on chromosome 22q13 including *MLC1*. No biological candidates are identified in this region, therefore further analyses may be needed to explain the causative mechanism for this association signal.

Among the novel WHR_adjBMI_ loci, rs116718588 is located between *TCF7L2* and *HABP2*. *TCF7L2* is the most significant type 2 diabetes locus in African Americans [36] and other populations [37]. However, rs116718588 was not in LD (r^2^ < 0.01 in AFR) with the reported type 2 diabetes associated variants. The second lead variant rs2472591 is located near *SPRYD7*, *DLEU2* and *TRIM13*. This locus was associated with height in previous GWAS [6], but rs2472591 was not associated with height in our study (*P* > 0.05), suggesting different variants in this locus regulate different measures of body size. In addition, a surrogate of rs2472591, rs790943, is a cis-eQTL for *TRIM13* [26] suggesting it may be the target gene. *TRIM13* encodes an E3 ubiquitin-protein ligase involved in endoplasmic reticulum-associated degradation. The third lead variant rs140858719 is located between *SSX2IP* and *LPAR3*. *LPAR3* is a plausible candidate as it encodes a receptor for lysophosphatidic acid (LPA). The autotaxin/LPA pathway mediates diverse biological actions including activation of preadipocyte proliferation [38], suppression of brown adipose differentiation [39], and promotion of systematic inflammation [40] which lead to increased risk for cardiometabolic diseases including obesity and insulin resistance [41–42]. LPA receptor 1 which is highly expressed in adipocytes and the gut primarily mediates these effects [43]. It has also been reported that LPA, via LPA1 and LPA3 receptors, mediated leukocytes recruitment and pro-inflammatory chemokine secretion during inflammation [44]. The fourth lead variant rs185693786 is located at intron 2 of *PDE3B*. The association signal spanned a large genomic region and harbors GWAS loci for adiponectin and height. Phosphodiesterase 3B is critical for mediating insulin/IGF-1 inhibition of cAMP signaling in adipocytes, liver, hypothalamus and pancreatic β cells [45]. *Pde3b*-knockout mice exhibited multiple alterations in regulation of lipolysis, lipogenesis, and insulin secretion, as well as signs of peripheral insulin resistance [46]. PDE3B expression has been reported to be higher in microvascular endothelial cell culture derived from skeletal muscles from male rats than in female rats [47]. The fifth lead variant rs6499129 is located intergenic between *ZDHHC1* and *HSD11B2*. *HSD11B2* encodes 11β-hydroxysteroid dehydrogenase type 2 which converts the active glucocorticoids to inactive metabolites. HSD2 activity was elevated in severe obesity and negatively associated with insulin sensitivity [48]. HSD2 expression is higher in omental than abdominal subcutaneous adipose tissue which may contribute to adipocyte hypertrophy and visceral obesity [49]. The sixth lead variant rs378854 is located at the long non-coding RNA *CASC8*. Associations of variants at *CASC8* have been reported for various cancers [50–52] but no association was reported for cardiometabolic traits.

In our SNP and locus transferability analyses, a moderate number of EA-derived BMI and WHR_adjBMI_ associated variants shared the same trait-raising alleles and displayed nominally significant associations in AA individuals, similar to previous findings [11–12]. While the BMI variants were similar in terms of their effect sizes and frequencies of trait-raising alleles between EA and AA populations, there were more discrepancies for WHR_adjBMI_ variants. In addition, a substantial proportion of lead regional variants in AA were not in strong LD with EA lead variants, suggesting AA populations either have different association signals or the results may be spurious. Taken together, only <30% of EA loci were associated with BMI and WHR_adjBMI_ in AA.

Trans-ethnic fine mapping improved resolution to refine putative causal variant(s) in some loci as compared to using EA studies alone. In the meta-analyses of AA and EA GWAS, four BMI loci (*SEC16B*, *STXBP6*, *FTO* and *MC4R*) and six WHR_adjBMI_ loci (*PPARG*, *ADAMTS9*, *SNX10*, *KLF13*, *CMIP* and *PEMT*) only contained one variant in the 99% credible sets. Among 16 BMI and 3 WHR_adjBMI_ loci that were examined in both the previous trans-ethnic meta-analysis studies using HapMap imputation [7–8] and the present study, the number of variants and the interval of credible sets were either the same or lower in the present study for 13 and 15 loci, respectively. The majority of credible variants are non-coding in those sets containing ≤ 20 variants. Several of them located at the *MTCH2*, *MAP2K5*, *NLRC3*, *ATP2A1*, *TBX15-WARS2* and *FAM13A* loci are cis-eQTL variants regulating nearby gene expression in subcutaneous and/or visceral adipose tissue, suggesting the putative causal variants may have a regulatory role instead of directly altering protein structure and function. Despite the low posterior probabilities, the coding changes of credible variants at *ADCY3* and *SH2B1* suggest that they may be the causal genes in the respective loci modulating BMI. Further studies are warranted to delineate putative causal variants including functional annotation in trans-ethnic fine mapping efforts [53].

Our large-scale GWAS meta-analyses in African ancestry individuals imputed to the 1000 Genomes reference panel, complemented by imputation of European GWAS using summary statistics and additional sex-stratified analyses, boosts the study power and improves resolution, leading to the identification of nine novel loci and fine mapping 37 loci with tractable credible sets. We observed significant associations for variants with MAF ≥ 0.5%, but rare variants were unlikely to be detected due to limited power and poor imputation quality. Large scale sequencing studies are needed to evaluate the contribution of rare variants in modulating complex traits such as BMI and WHR. Given the substantially larger sample size in European than in African ancestry samples, the trans-ethnic fine mapping results are largely driven by variants showing strong associations in Europeans. Future trans-ethnic studies including additional non-European populations will further improve the fine mapping effort.

## Materials and methods

### Study design

We used a three-stage design to evaluate genetic associations with BMI and WHR_adjBMI_ in sex-combined and sex-stratified samples (S1 Fig). Stage 1 included GWAS meta-analyses in AA individuals and stage 2 included replication of top associations from stage 1. Stage 3 included meta-analysis of top associations from stages 1 and 2 AA studies and EA meta-analysis results. In the discovery stage 1 of AAAGC, 17 GWAS of up to 42,752 AA individuals (16,559 men and 26,193 women; 41,696 African Americans and 1,056 Africans) were included for the BMI analyses. A total of 10 GWAS of up to 20,384 AA individuals (4,783 men and 15,601 women; all African Americans) were included for the WHR_adjBMI_ analyses. For variants with *P* < 1×10^−4^ in either the sex-combined or the sex-stratified meta-analyses, stage 2 replication was performed in additional AA individuals from AAAGC (N = 10,143 for BMI, N = 2,711 for WHR_adjBMI_), followed by meta-analysis with EA individuals from the GIANT consortium (322,154 for BMI, 210,086 for WHR_adjBMI_). Variants that reached genome-wide significance (*P* < 5×10^−8^) were assessed for associations with BMI in two cohorts of children (N = 7,222). All AA participants in these studies provided written informed consent for the research, and approval for the study was obtained from the ethics review boards at all participating institutions. Detailed descriptions of each participating study and measurement and collection of height, weight, waist and hip circumferences are provided in S1 Text, S1 and S2 Tables.

### Genotyping, imputation and quality control

Genotyping in each study was performed with Illumina or Affymetrix genome-wide SNP arrays. Pre-phasing and imputation of missing genotypes in each study was performed using MaCH/ minimac [20] or SHAPEIT2/IMPUTEv2 [21–22] using the 1000 Genomes Project cosmopolitan reference panel (Phase I Integrated Release Version 3, March 2012) [18]. The details of the array, genotyping and imputation quality-control procedures and sample exclusions for each study are listed in S3 Table. In general, samples reflecting duplicates, low call rates, gender mismatch, or population outliers were excluded. Variants were excluded by the following criteria: call rate < 0.95, minor allele count (MAC) ≤ 6, Hardy-Weinberg Equilibrium (HWE) *P* < 1×10^−4^, imputation quality score < 0.3 for minimac or < 0.4 for IMPUTE, or absolute allele frequency difference > 0.3 compared with expected allele frequency (calculated as 1000 Genomes frequency of AFR × 0.8 + EUR × 0.2).

### Performance of 1000 Genomes imputation in African ancestry

We evaluated the performance of 1000 Genomes imputation using the largest study, the Women’s Health Initiative (WHI) (N = 8,054). A total of 25.1 million variants with MAF ≥ 0.1% were imputed to the 1000 Genomes reference panel. Of these, 98.1% (8.8 million) common variants, 95.4% (9.3 million) low frequency variants (0.5% ≤ MAF < 5%), and 72.5% (4.6 million) rare variants (0.1% ≤ MAF < 0.5%) were well imputed with IMPUTE info scores ≥ 0.3 (S4 Table). Notably, these frequencies are slightly lower than those obtained by imputation using 1000 Genomes phase 1 interim reference panel in Europeans [54]. However, 72.6%, 95.5% and 99.5% of the common, low frequency and rare variants, respectively, from the 1000 Genomes reference panel were not present in the HapMap and therefore demonstrate deeper coverage of the genome, particularly for the low frequency and rare variants.

### Study-level association analyses

At all stages, genome-wide association analyses were performed by each of the participating studies. BMI was regressed on age, age squared, principal components and study site (if needed) to obtain residuals, separately by sex and case-control status, if needed. WHR was regressed on age, age squared, principal components, BMI and study site to obtain residuals, separately by sex and case-control status. Principal components were included to adjust for admixture proportion and population structure within each study. Residuals were inverse-normally transformed to obtain a standard normal distribution with mean of zero and standard deviation of one. For studies with unrelated subjects, each variant was tested assuming an additive genetic model with each trait by regressing the transformed residuals on the number of copies of the variant effect allele. The analyses were stratified by sex and case-control status (if needed). For studies that included related individuals, family based association tests were conducted that took into consideration the genetic relationships among the individuals. Sex stratified, case-control stratified and combined analyses were performed. Association results with extreme values (absolute beta coefficient or standard error ≥ 10), primarily due to small sample sizes and/or low minor allele count, were excluded for meta-analysis.

### Imputation of European GWAS summary statistics to 1000 Genomes

The latest summary statistics of sex-combined and sex-stratified meta-analyses of BMI and WHR_adjBMI_ imputed to the HapMap reference panel in EA from the Genetic Investigation of ANthropometric Traits (GIANT) consortium were obtained from http://www.broadinstitute.org/collaboration/giant/index.php/GIANT_consortium_data_files [7–8]. These association summary statistics were used to impute z-scores of unobserved variants at the 1000 Genomes Project EUR reference panel (Phase I Integrated Release Version 3) using the ImpG program [23]. In brief, palindromic variants (AT/CG) and variants with allele mismatch with the reference were removed from the data. Using the ImpG-Summary method, the z-score of an unobserved variant was calculated as a linear combination of observed z-scores weighted by the variance-covariance matrix between variants induced by LD within a 1 Mb window from the reference haplotypes. The sample size of each unobserved variant was also interpolated from the sample sizes of observed variants using the same weighting method for z-score as $N_{i}=\sum_{t=1}^{t=T}\frac{\left|w_{i,t}\right|}{\sum \left|w_{i,t}\right|}N_{t}$. Here, t = 1,2,….,T, where T is the number of observed variants, *w*_*i*,*t*_ is the element of the covariance matrix Σ_*i*,*t*_ for the unobserved variant *i* and the observed variant *t* within window. The performance of imputation was assessed by *r*^*2*^*pred*, with similar characteristics as the standard imputation accuracy metric *r*^*2*^*hat* [20]. Results of variants with *r*^*2*^*pred* ≥ 0.6 were used in subsequent analyses.

### Meta-analysis

In the discovery stage 1, association results were combined across studies in sex-combined and sex-stratified samples using inverse-variance weighted fixed-effect meta-analysis implemented in the program METAL [55]. The study-specific λ values of association ranged from 0.97 to 1.05 for BMI, and 0.98 to 1.05 for WHR_adjBMI_ (S3 Table). Genomic control correction [56] was applied to each study before meta-analysis, and to the overall results after meta-analysis (λ = 1.07 for BMI, 1.01 for WHR_adjBMI_). Variants with results generated from < 50% of the total sample size for each trait were excluded. After filtering, the numbers of variants reported in the meta-analyses were 17,972,087 for BMI, and 20,502,658 for WHR_adjBMI_.

Variants with *P* < 1×10^−4^ in stage 1 sex-combined or sex-stratified meta-analyses were carried forward for replication in additional AA individuals (stage 2) and EA individuals (stage 3). For each of the replication AA studies, trait transformation and association were performed as in stage 1 and results were meta-analyzed using the inverse-variance method in METAL. For the replication study in EA, HapMap imputed summary statistics of each trait from the GIANT consortium were used to impute z-scores of unobserved variants at the 1000 Genomes.

In stages 1 and 2, meta-analysis results of AA studies were combined using the inverse-variance weighted method. In all stages including both AA and EA studies, meta-analysis results expressed as signed z-scores were combined using the fixed effect sample size weighted method in METAL due to the lack of beta and standard error estimates from the ImpG program [23]. Evidence of heterogeneity of allelic effects between males and females, within and across stages were assessed by the *I*^*2*^ statistic in METAL. Genome-wide significance was declared at *P* < 5×10^−8^ from each of the sex-combined and sex-stratified meta-analysis including AA and/or combined AA and EA individuals. Difference in effects between men and women was assessed using Cochran’s Q test and nominal *P*_*het*_ < 0.05 declared as significant. A lead variant in a locus was defined as the most significant variant within a 1 Mb region. A novel locus was defined as a lead variant with distance > 500 kb from any established lead variants reported in previous studies. By convention, a locus was named by the closest gene(s) to the lead variant.

### Conditional and joint analyses of summary statistics

For the genome-wide significant loci identified in sex-combined and sex-stratified analyses in AA (stages 1+2), we used the program GCTA [57–58] to select the top independent associated variants from summary statistics of the meta-analyses. This method uses the LD correlations between variants estimated from a reference sample to perform an approximate conditional association analysis. We used 8,054 unrelated individuals of African ancestry from the WHI cohort with ~15.7M variants available as the reference sample for LD estimation. To select the top independent variants in the discovery and replication meta-analysis results, we first selected all variants that had *P* < 5×10^−8^ and conducted analysis conditioning on the selected variants to search for the top variants iteratively via a stepwise model to select the independent variants from this list. Then we proceeded to condition the rest of the variants that had *P* > 5×10^−8^ on the list of independent variants in the same fashion until no variant had conditional *P* that passed the significance level *P* < 5×10^−8^. Finally, all the selected variants were fitted jointly in the model for effect size estimation.

We also tested if the genome-wide significant variants identified from sex-combined GWAS in AA and the locus-wide significant variants identified from sex-combined and sex-specific locus transferability studies in AA were independent from nearby established loci identified from EA studies [7–8]. First, the published lead variants from EA studies were used to search for all surrogate variants that were in high LD (r^2^>0.8 in 1000 Genomes Project EUR population). Second, these variants were pruned to select only variants in low LD in AA (r^2^<0.3 in the 1000 Genomes Project AFR population) to avoid collinearity in conditional analysis. Third, association analysis was conducted on the AA significant variants conditioned on the selected EA lead and surrogate variants, using the program GCTA and estimated LD correlation from the WHI cohort. For genome-wide significant loci, an AA derived association signal is considered as independent from the established EA signals when the difference in–log*P* <3 and difference in effect size < 1 standard error after conditional analysis. For locus-wide significant loci, given the lower level of significance, independence is only considered as difference in effect size < 1 standard error after conditional analysis.

### SNP and locus transferability analyses

We investigated the transferability of EA BMI and WHR associated variants and loci in AA individuals from stage 1 sex-combined and sex-stratified meta-analyses. First, we tested for replication of lead variants previously reported to be associated with BMI (176 variants from 170 loci) and WHR_adjBMI_ (84 variants from 65 loci) at genome-wide significance in sex-combined and sex-stratified analyses from the GIANT consortium studies [7–9]. We defined SNP transferability as an EA lead variant sharing the same trait-raising allele at nominal *P* < 0.05 in AA individuals. To account for differences in local LD structure across populations, we also interrogated the flanking 0.1cM regions of the lead variants to search for the best variants with the smallest association *P* in AA individuals. Locus-wide significance was declared as *P*_*locus*_ < 0.05 by Bonferroni correction for the effective number of tests within a locus, estimated using the Li and Ji approach [59].

### Fine mapping analyses

We compared the credible set intervals of established loci that showed locus-wide significance (*P*_*locus*_ < 0.05) in the sex-combined or sex-specific analyses from this study in summary statistics datasets including the 1000 Genomes imputed results from GIANT, AAAGC and meta-analysis of GIANT and AAAGC. In each dataset, a candidate region is defined as the flanking 0.1cM region of the lead variant reported by the GIANT consortium. Under the assumption of one causal variant in a region of *M* variants, the posterior probability of a variant *j* with association statistics Z driving the association, *P*(*C*_*j*_|*Z*), was calculated using the formula $P(C_{j}|Z)=\frac{\exp(\frac{1}{2}z_{j}^{2})}{\sum_{j=1}^{M}exp(\frac{1}{2}z_{j}^{2})}$. A 99% credible set was constructed by ranking all variants by their posterior probability, followed by adding variants until the credible set has a cumulative posterior probability > 0.99 [53].

### Bioinformatics

#### Functional annotation of novel variants

To determine whether any of our nine novel GWAS lead variants identified in the sex-combined and sex-specific analyses might be tagging potentially functional variants, we identified all variants within 1 Mb and in LD (r^2^ > 0.7, 1000 Genomes AFR) with our lead variants. As such, we identified 137 variants and annotated each of them using ANNOVAR [60]. The predicted functional impact for coding variants were assessed via the Exome Variant Server (http://evs.gs.washington.edu/EVS/) for PhastCon, GERP [61], and PolyPhen [62], as well as SIFT [63].

We further characterized the variants that were in LD with the novel variants using the web-based tool RegulomeDB (http://regulomedb.org/) [24]. The variants that were likely to affect binding and linked to expression of a gene target (scores 1a-1f) based on “eQTL, transcription factor (TF) binding, matched TF motif, matched DNase footprint and DNase peak” or were only likely to affect binding (scores 2a-2c) based on “TF binding, matched TF motif, matched DNase footprint and DNase peak” were selected. For these variants, the sequence conservation (GERP and SiPhy [64]), the epigenomic data from the Roadmap Epigenomic project (ChromHMM states corresponding to enhancer or promoter elements, histone modification ChIP-seq peaks, and DNase hypersensitivity data peaks), the regulatory protein binding from the ENCODE project, the regulatory motifs based on commercial, literature and motif-finding analysis of the ENCODE project, and the eQTLs from Genotype-Tissue Expression (GTEx) project [65] were extract from web-based HaploReg v4 [25]. For variants within the tractable credible sets in the fine mapping analyses, similar analyses were also conducted.

#### Cross-trait associations

To assess whether the novel loci identified in the sex-combined and sex-specific analyses were associated with any related cardiometabolic and anthropometric traits, or may be in high LD with known eQTLs, we examined the NHGRI-EBI GWAS Catalog [27] and the GRASP (Genome-Wide Repository of Associations Between SNPs and Phenotypes) catalog [28] for reported variant-trait associations near our lead variants. We supplemented the catalogs with additional genome-wide significant associations of interest from the literature [7–9,66]. We used PLINK to identify variants within 1 Mb of lead variants. All variants within the specified regions with r^2^ > 0.7 (1000 Genomes AFR) were retained from the catalogs for further evaluation.

### Power analysis

Given our sample sizes in the discovery and replication stages in our African ancestry populations, we have >80% power to detect variants explaining 0.08% variance for BMI that corresponds to effect sizes of 0.09 and 0.20 SD units for MAF of 0.05 and 0.01, respectively. For WHR_adjBMI_, we have >80% power to detect variants explaining 0.18% variance that corresponds to effect sizes of 0.14 and 0.30 SD units for MAF of 0.05 and 0.01, respectively.

## Supporting information

S1 Fig Study design of GWAS meta-analyses and replications for BMI and WHR_adjBMI_. (PDF)

S2 Fig Quantile-quantile plot of 1000 genomes phase 1 imputed discovery results and their associations to adult BMI in men and women of African ancestry using all variants and only variants outside of known GWAS loci. (PDF)

S3 Fig Quantile-quantile plot of 1000 genomes phase 1 imputed discovery results and their associations to adult BMI in women only and men only of African ancestry using all variants and only variants outside of known GWAS loci. (PDF)

S4 Fig Quantile-quantile plot of 1000 genomes phase 1 imputed discovery results and their associations to adult waist-to-hip ratio adjusted for BMI (WHR_adjBMI_) in men and women of African ancestry using all variants and only variants outside of known GWAS loci. (PDF)

S5 Fig Quantile-quantile plot of 1000 genomes phase 1 imputed discovery results and their associations to adult waist-to-hip ratio adjusted for BMI (WHR_adjBMI_) in women only and men only of African ancestry using all variants and only variants outside of known GWAS loci. (PDF)

S6 Fig Manhattan plot of 1000 genomes phase 1 imputed discovery results and their associations to adult BMI in men and women of African ancestry. (PDF)

S7 Fig Manhattan plot of 1000 genomes phase 1 imputed discovery results and their associations to waist-to-hip ratio adjusted for BMI (WHR_adjBMI_) in men and women of African ancestry. (PDF)

S8 Fig Miami plot of 1000 genomes phase 1 imputed discovery results and their associations to adult BMI in women only (top) and men only (bottom) of African ancestry. (PDF)

S9 Fig Miami plot of 1000 genomes phase 1 imputed discovery results and their associations to adult waist-to-hip ratio adjusted for BMI (WHR_adjBMI_) in women only (top) and men only (bottom) of African ancestry. (PDF)

S10 Fig Locuszoom plots using discovery results for established loci that reached genome-wide significance: (A) *SEC16B*, *TMEM18*, *GNPDA2*, *GALNT10*, *KLHL32*, *FTO* and *MC4R* for BMI in men and women combined; and (B) *ADAMTS9-AS2* for waist-to-hip ratio adjusted for BMI (WHR_adjBMI_) in men and women combined. All plots use AFR LD from the 1000 Genomes phase 1 reference panel. In each plot, the most significant variant within a 1Mb regional locus is highlighted. *P*-values for all variants including the most significant variant are based on the African ancestry discovery phase only (AA-Discovery). In addition, for the most significant variant, *P*-values are annotated and illustrated from the African ancestry discovery and replication phases (AA-Discovery+Replication).
(PDF)

S11 Fig Correlation of effect sizes for (A) BMI and (B) WHR_adjBMI_, and effect allele frequencies for (C) BMI and (D) WHR_adjBMI_ in European and African ancestry studies in SNP transferability analyses.
(PDF)

S1 Table Study design and sample quality control of discovery and replication studies. (XLSX)

S2 Table Study-specific descriptive statistics of discovery and replication studies. (XLSX)

S3 Table Genotyping methods, quality control of variants, imputation, and statistical analysis in discovery and replication studies. (XLSX)

S4 Table Comparison of coverage of variants using the 1000 Genomes and HapMap reference panels for imputation in the Women's Health Initiative study. (XLSX)

S5 Table Comparison of lead variants between 1000 Genomes and HapMap imputed meta-analysis in AA in previously identified BMI loci in discovery and replication studies. (XLSX)

S6 Table Associations of lead variants from novel and previously identified BMI and WHR_adjBMI_ loci in combined and sex-stratified analyses of African ancestry discovery and replication samples. (XLSX)

S7 Table Association of African Ancestry sex-combined genome-wide significant variants in children of African ancestry. (XLSX)

S8 Table Putative coding or regulatory variants in linkage disequilibrium (r^2^ > 0.7) with WHR_adjBMI_ loci. (XLSX)

S9 Table Previously-reported associations of novel BMI and WHR_adjBMI_ loci with other traits in the GRASP Catalog. This table lists all previously-reported associations within 1 Mb (+/- 500 kb) and in high LD (r^2^ >0.7) with our lead novel SNPs along with relevant annotation (e.g. miRNA target binding site, variant location relevant to nearest gene, gene function prediction) reported in the GRASP Catalog.
(XLSX)

S10 Table Conditional analysis of African ancestry primary and secondary lead SNPs with European lead SNPs in previously identified BMI and WHR_adjBMI_ loci. (XLSX)

S11 Table SNP transferability of BMI and WHR_adjBMI_ lead SNPs from European sex combined and sex stratified GWAS in African ancestry individuals. (XLSX)

S12 Table Locus transferability and conditional analyses of European BMI and WHR_adjBMI_ loci in African ancestry individuals. (XLSX)

S13 Table Fine mapping of novel loci and previously identified loci with locus-wide significance in African ancestry individuals using 1000 Genomes imputed results from African, European and combined ancestries. (XLSX)

S14 Table Functional characterization of variants in tractable credible sets in meta-analysis of African and European ancestry GWAS. (XLSX)

S1 Text Supplementary note. (DOCX)

S2 Text Members of the BMDCS Group. (DOCX)
