# Supplementary figures and images for: Discovery and fine-mapping of adiposity loci using high density imputation of genome-wide association studies in individuals of African ancestry: African Ancestry Anthropometry Genetics Consortium
Source: PLoS Genet. 2017 Apr 21;13(4):e1006719. doi: 10.1371/journal.pgen.1006719 (PMC5419579; doi:10.1371/journal.pgen.1006719)

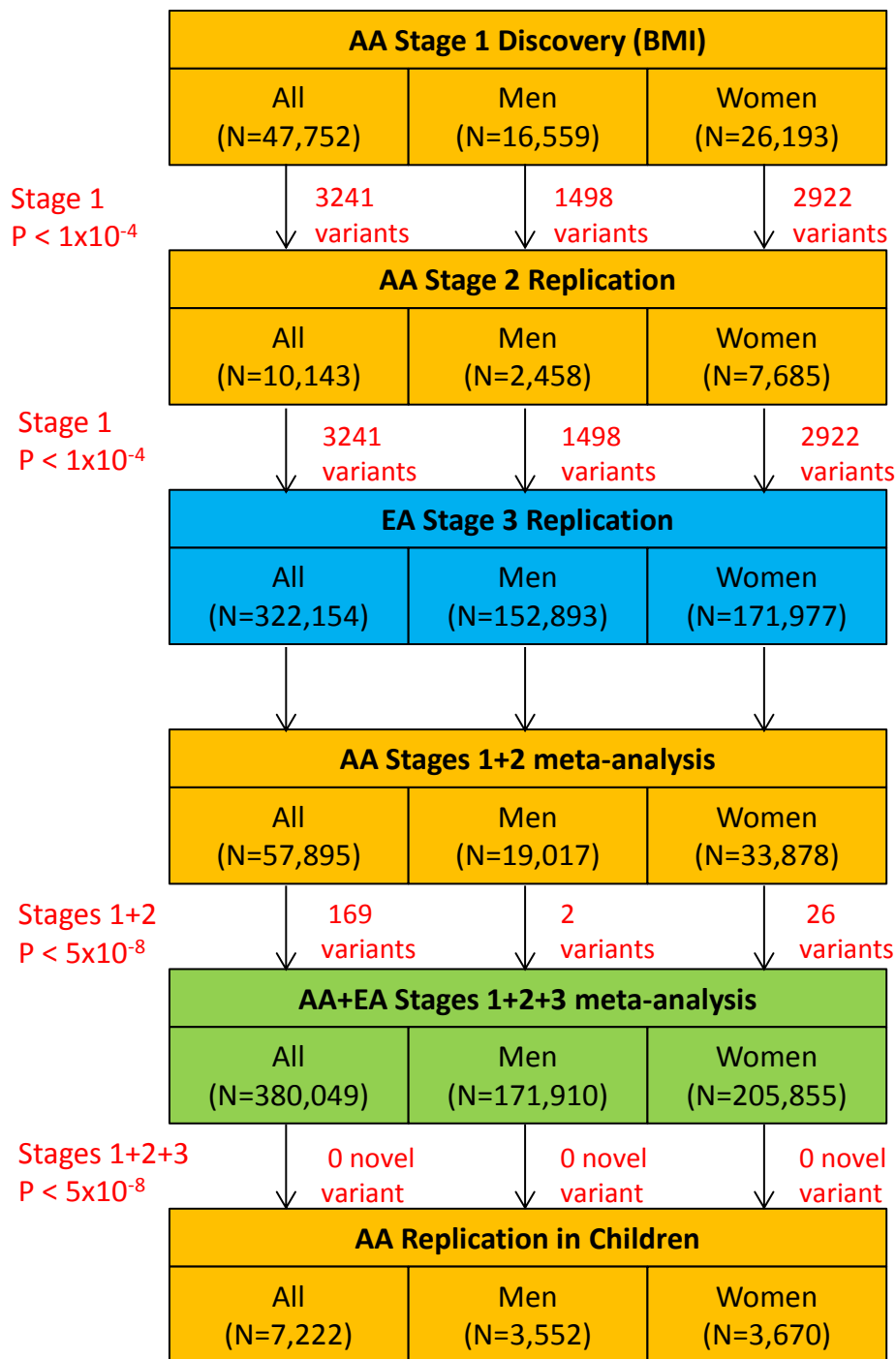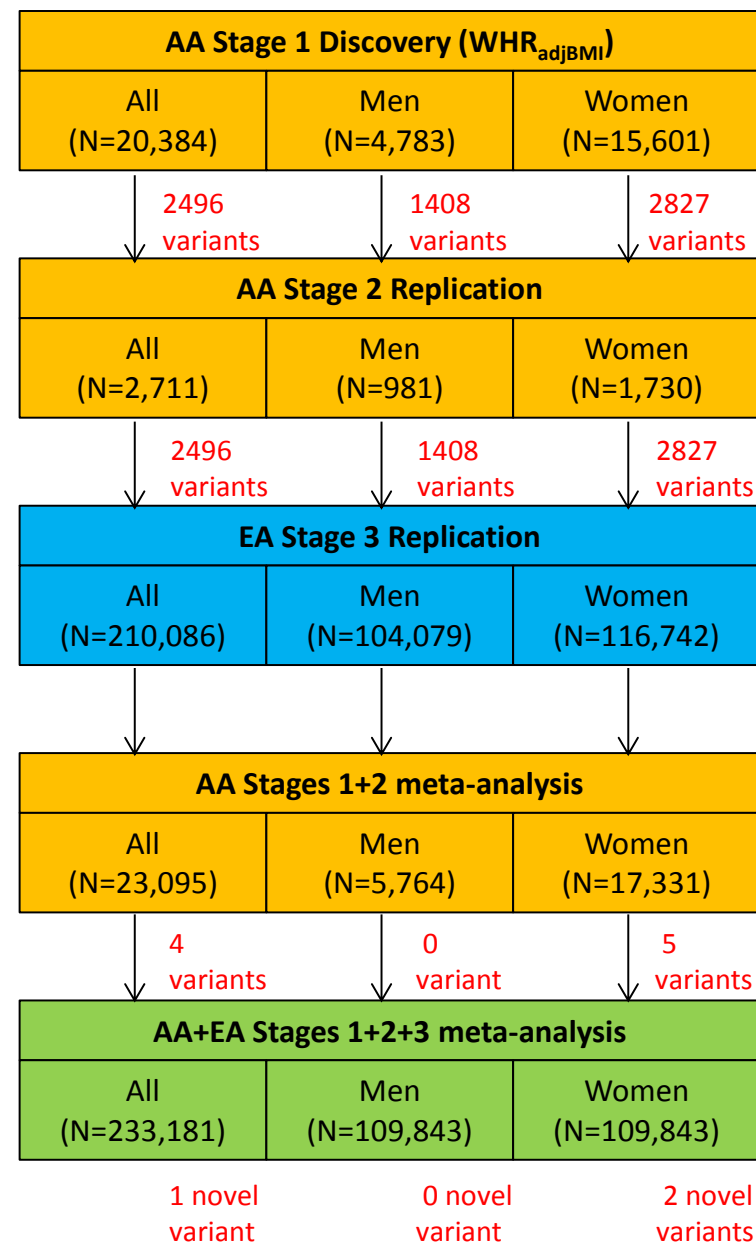

**AA: African ancestry**  
**EA: European ancestry**

Supplement: S1 Fig — (PDF) [file pgen.1006719.s001.pdf]

# BMI, Men and Women Combined

Known loci

Novel SNPs, Pvalue<5e-08

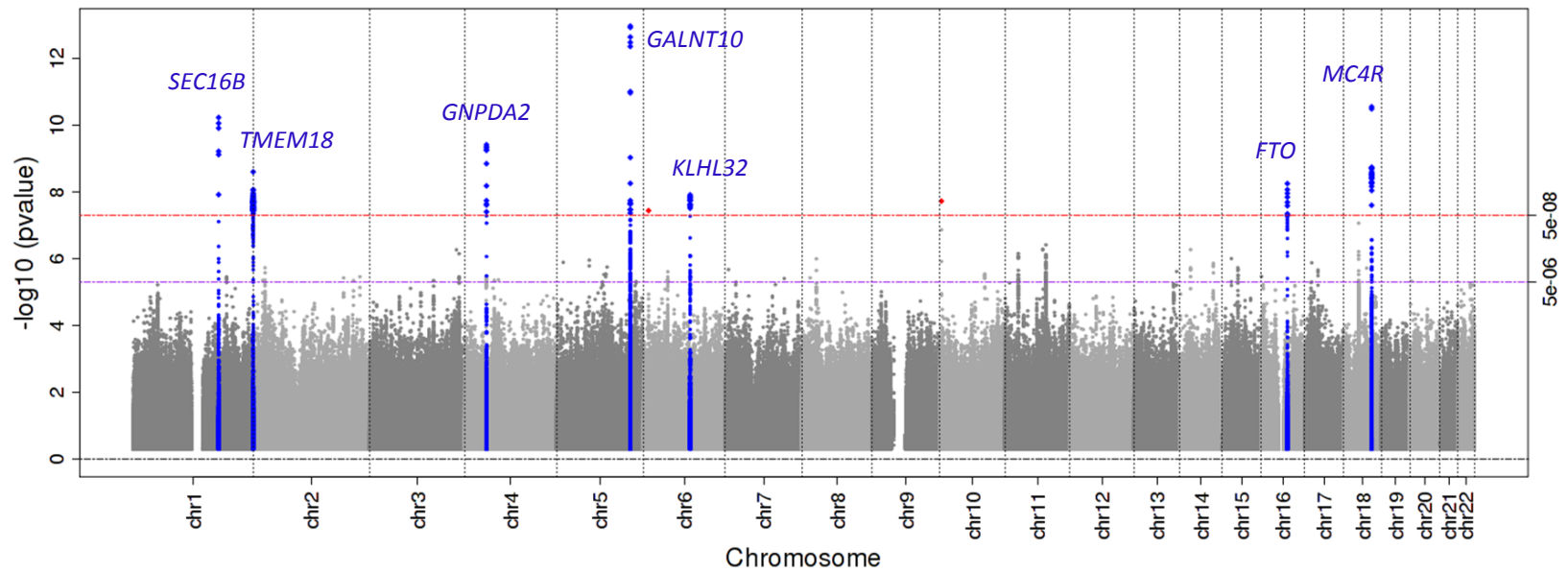

Supplement: S6 Fig — (PDF) [file pgen.1006719.s006.pdf]
