# Supplementary material for: Discovery and fine-mapping of adiposity loci using high density imputation of genome-wide association studies in individuals of African ancestry: African Ancestry Anthropometry Genetics Consortium
Source: PLoS Genet. 2017 Apr 21;13(4):e1006719. doi: 10.1371/journal.pgen.1006719 (PMC5419579; doi:10.1371/journal.pgen.1006719)
Supplement: S4 Fig — (PDF) [file pgen.1006719.s004.pdf]

WHRadjBMI, Men and Women Combined

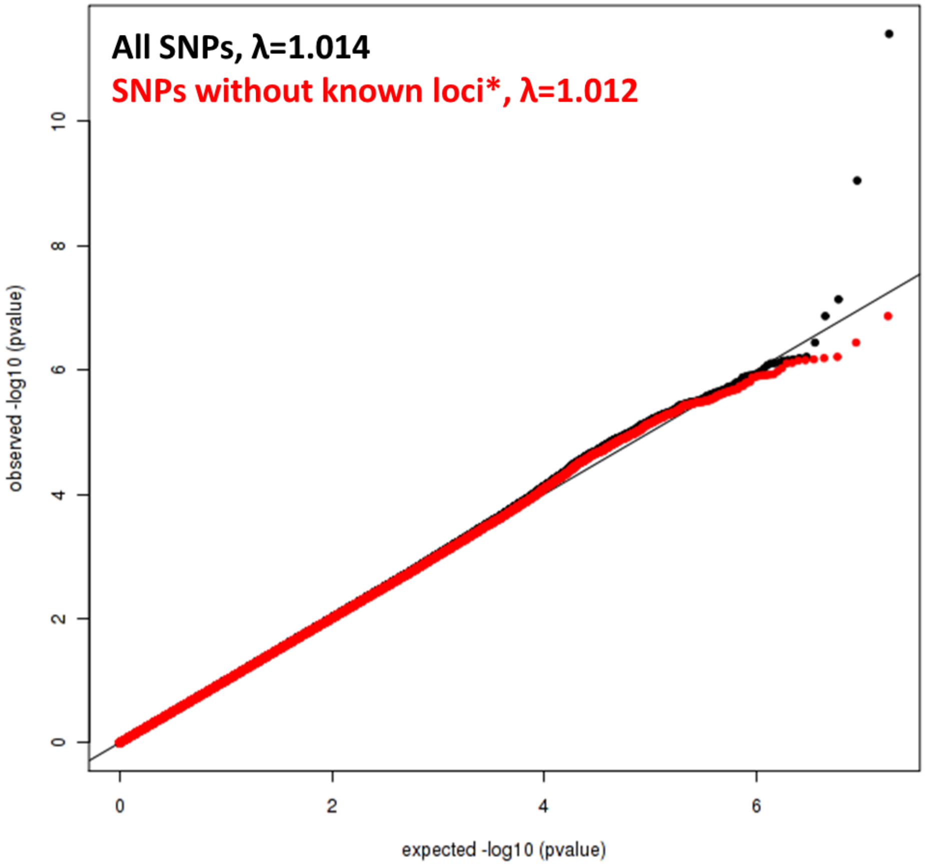

*Known loci removed are defined by +/- 500kb around all lead SNPs reported in the literature.
