# Supplementary material for: Discovery and fine-mapping of adiposity loci using high density imputation of genome-wide association studies in individuals of African ancestry: African Ancestry Anthropometry Genetics Consortium
Source: PLoS Genet. 2017 Apr 21;13(4):e1006719. doi: 10.1371/journal.pgen.1006719 (PMC5419579; doi:10.1371/journal.pgen.1006719)
Supplement: S7 Fig — (PDF) [file pgen.1006719.s007.pdf]

WHRadjBMI, Men and Women Combined

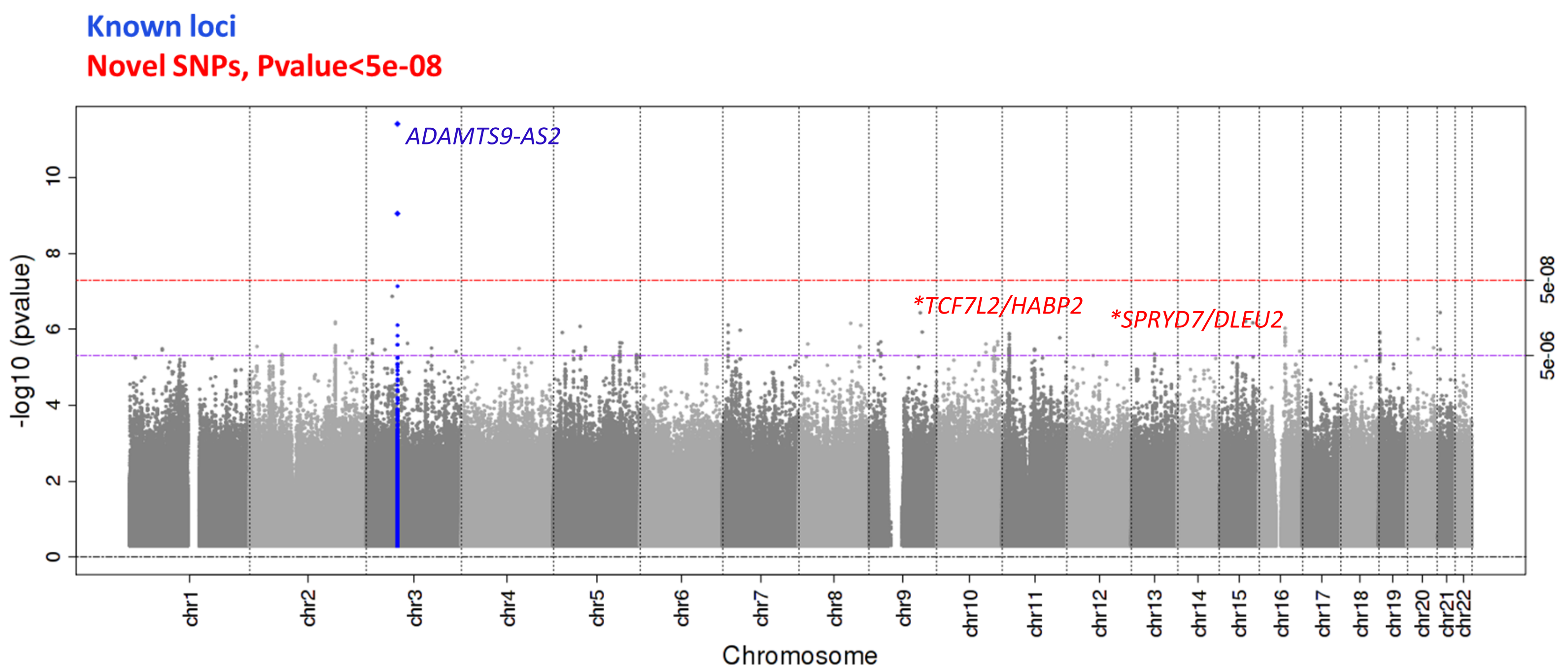

**TCF7L2/HABP2 (*chromosome 10, position 115,189,239) *and SPRYD7/DLEU2* (chromosome 13, position 50,536,360) were genome-wide significant after combining discovery with replication results.
