# Supplementary material for: Discovery and fine-mapping of adiposity loci using high density imputation of genome-wide association studies in individuals of African ancestry: African Ancestry Anthropometry Genetics Consortium
Source: PLoS Genet. 2017 Apr 21;13(4):e1006719. doi: 10.1371/journal.pgen.1006719 (PMC5419579; doi:10.1371/journal.pgen.1006719)
Supplement: S8 Fig — (PDF) [file pgen.1006719.s008.pdf]

BMI, Women only (top) and Men only (bottom)

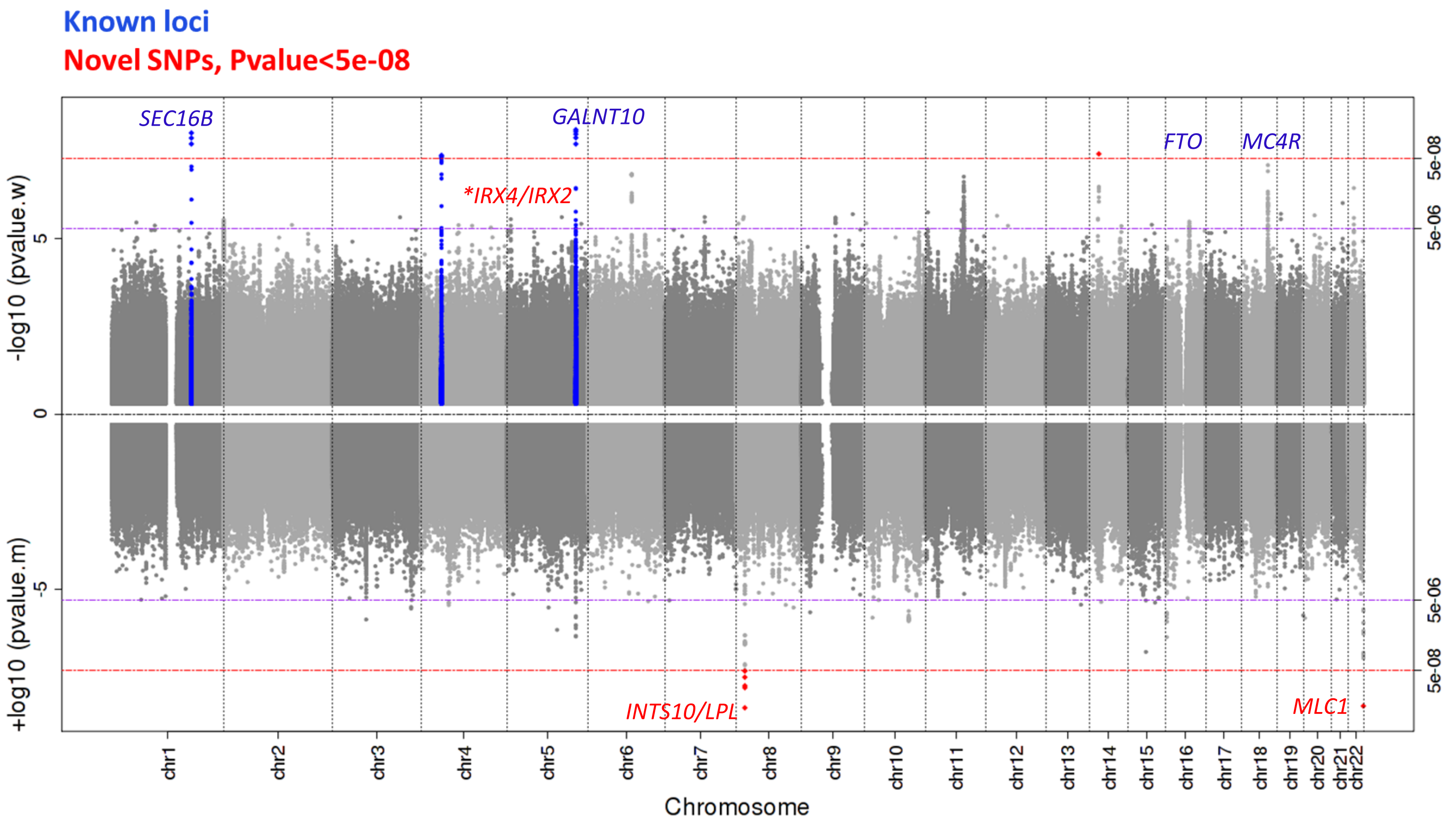

* *IRX4/IRX2* (chromosome 5, position 2,177,693), *FTO* (chromosome 16, position 53,828,066), and *MC4R* (chromosome 18, position 57,829,135) were genome-wide significant after combining discovery with replication results.
