# Supplementary material for: Discovery and fine-mapping of adiposity loci using high density imputation of genome-wide association studies in individuals of African ancestry: African Ancestry Anthropometry Genetics Consortium
Source: PLoS Genet. 2017 Apr 21;13(4):e1006719. doi: 10.1371/journal.pgen.1006719 (PMC5419579; doi:10.1371/journal.pgen.1006719)
Supplement: S9 Fig — (PDF) [file pgen.1006719.s009.pdf]

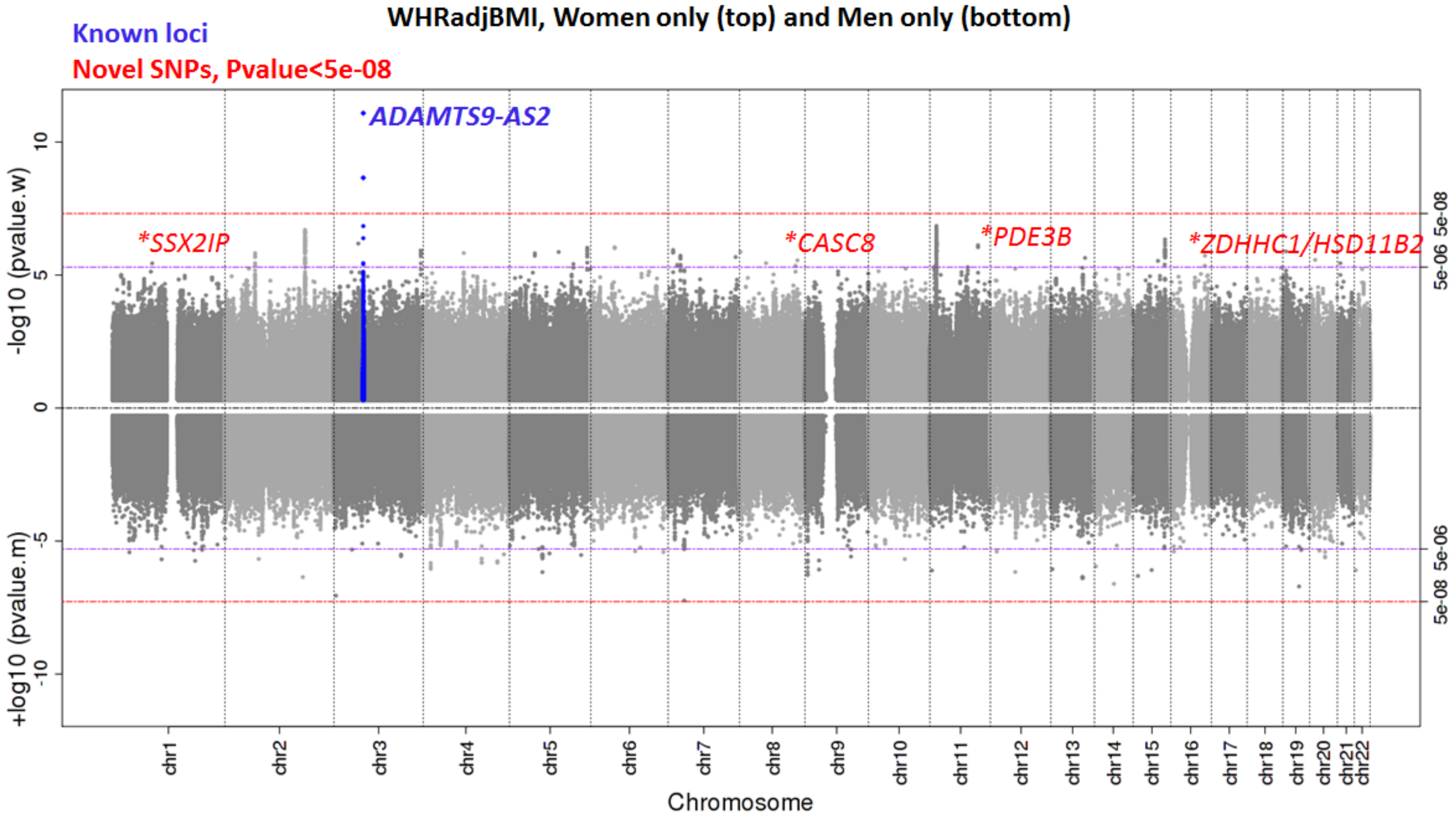

* *SSX2IP* (chromosome 1, position 85,203,061) and *PDE3B* (chromosome 11, position 14,804,296) were genome-wide significant after combining discovery with replication results. *CASC8* (chromosome 8, position 128323819) and *ZDHHC1/HSD11B2* (chromosome 16, position 67,458,251) were genome-wide significant after combining discovery with replication results and with results from the GIANT consortium. (European American data from Shungin D*, Winkler TW*, Croteau-Chonka DC*, Ferreira T*, Locke AE*, Magi R*, Strawbridge R, Pers TH, Fischer K, Justice AE, Workalemahu T, Wu JM, et al. (2015) New genetic loci link adipose and insulin biology to body fat distribution. Nature **518**, 187-196.)
