# Supplementary material for: Discovery and fine-mapping of adiposity loci using high density imputation of genome-wide association studies in individuals of African ancestry: African Ancestry Anthropometry Genetics Consortium
Source: PLoS Genet. 2017 Apr 21;13(4):e1006719. doi: 10.1371/journal.pgen.1006719 (PMC5419579; doi:10.1371/journal.pgen.1006719)
Supplement: S10 Fig — Locuszoom plots using discovery results for established loci that reached genome-wide significance: (A) SEC16B, TMEM18, GNPDA2, GALNT10, KLHL32, FTO and MC4R for BMI in men and women combined; and (B) ADAMTS9-AS2 for waist-to-hip ratio adjusted for BMI (WHRadjBMI) in men and women combined. All plots use AFR LD from the 1000 Genomes phase 1 reference panel. In each plot, the most significant variant within a 1Mb regional locus is highlighted. P-values for all variants including the most significant variant are based on the African ancestry discovery phase only (AA-Discovery). In addition, for the most significant variant, P-values are annotated and illustrated from the African ancestry discovery and replication phases (AA-Discovery+Replication). (PDF) [file pgen.1006719.s010.pdf]

(A)

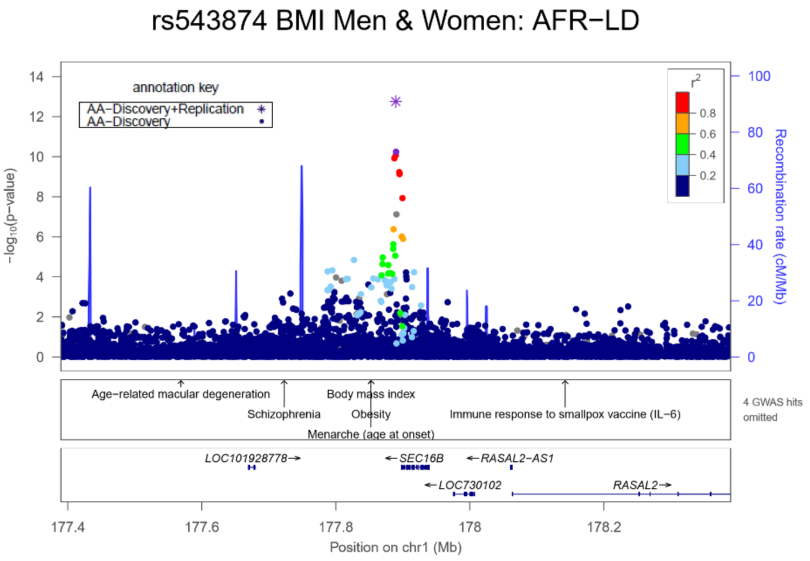

rs543874 BMI Men & Women: AFR−LD
annotation key
AA−Discovery+Replication
AA−Discovery
−log10(p−value)
Recombination rate (cM/Mb)
Age−related macular degeneration
Schizophrenia
Body mass index
Obesity
Menarche (age at onset)
Immune response to smallpox vaccine (IL−6)
4 GWAS hits omitted
LOC101928778
SEC16B
RASAL2−AS1
LOC730102
RASAL2
Position on chr1 (Mb)

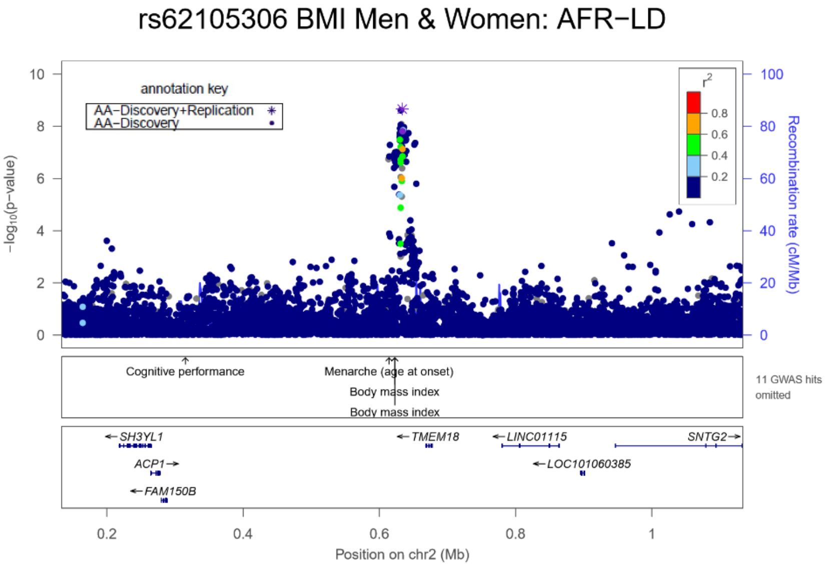

rs62105306 BMI Men & Women: AFR−LD
annotation key
AA−Discovery+Replication
AA−Discovery
−log10(p−value)
Recombination rate (cM/Mb)
Cognitive performance
Menarche (age at onset)
Body mass index
Body mass index
11 GWAS hits omitted
SH3YL1
ACP1
FAM150B
TMEM18
LINC01115
LOC101060385
SNTG2
Position on chr2 (Mb)

rs10938397 BMI Men & Women: AFR−LD

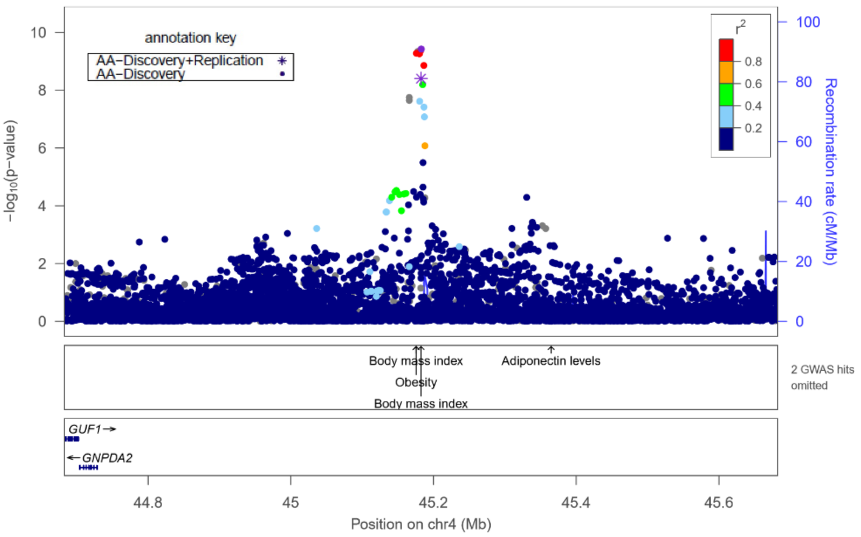

rs7708584 BMI Men & Women: AFR−LD

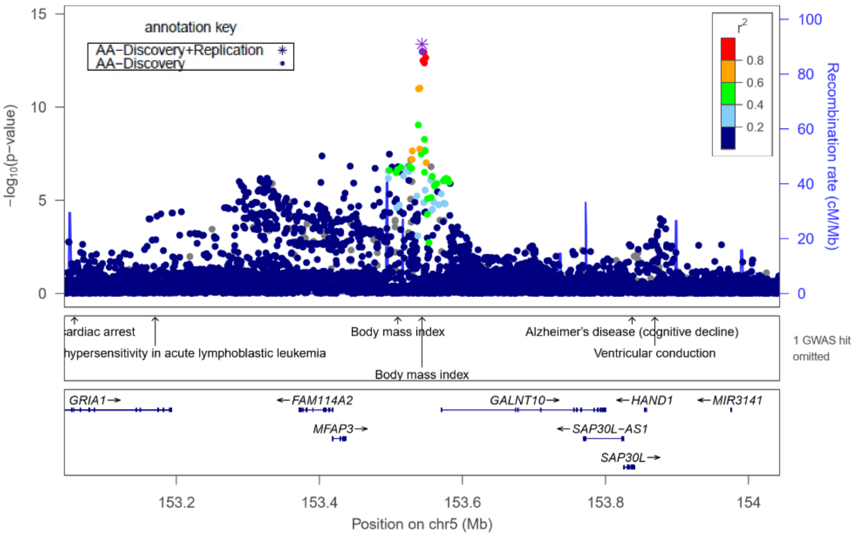

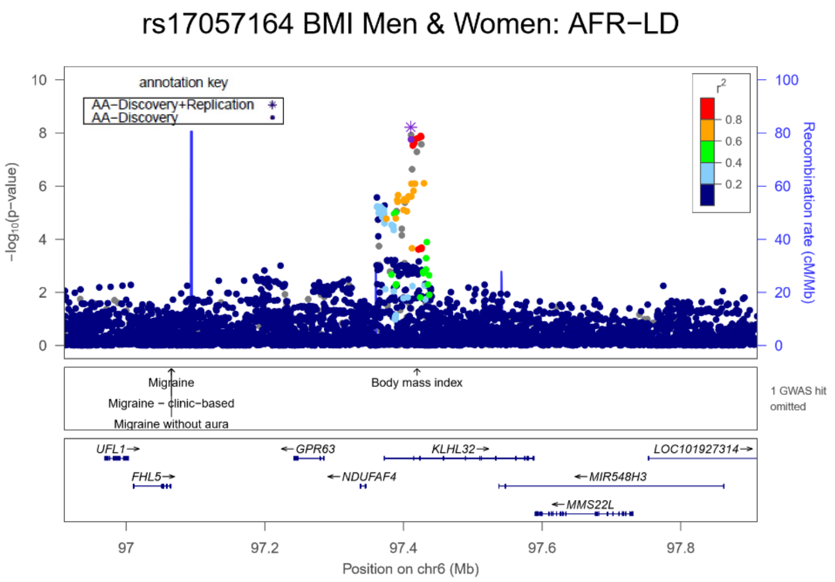

rs17057164 BMI Men & Women: AFR−LD
annotation key
AA−Discovery+Replication
AA−Discovery
−log10(p−value)
Recombination rate (cM/Mb)
Migraine
Migraine − clinic−based
Migraine without aura
Body mass index
1 GWAS hit omitted
UFL1
FHL5
GPR63
NDUFAF4
KLHL32
MIR548H3
MMS22L
LOC101927314
Position on chr6 (Mb)

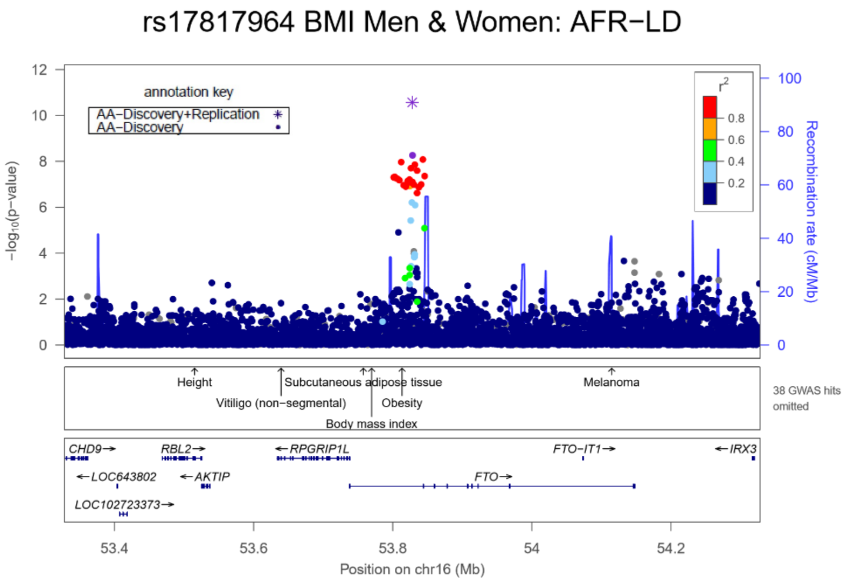

rs17817964 BMI Men & Women: AFR−LD
annotation key
AA−Discovery+Replication
AA−Discovery
−log10(p−value)
Recombination rate (cM/Mb)
Height
Vitiligo (non−segmental)
Subcutaneous adipose tissue
Body mass index
Obesity
Melanoma
38 GWAS hits omitted
CHD9
LOC643802
LOC102723373
RBL2
AKTIP
RPGRIP1L
FTO
FTO−IT1
IRX3
Position on chr16 (Mb)

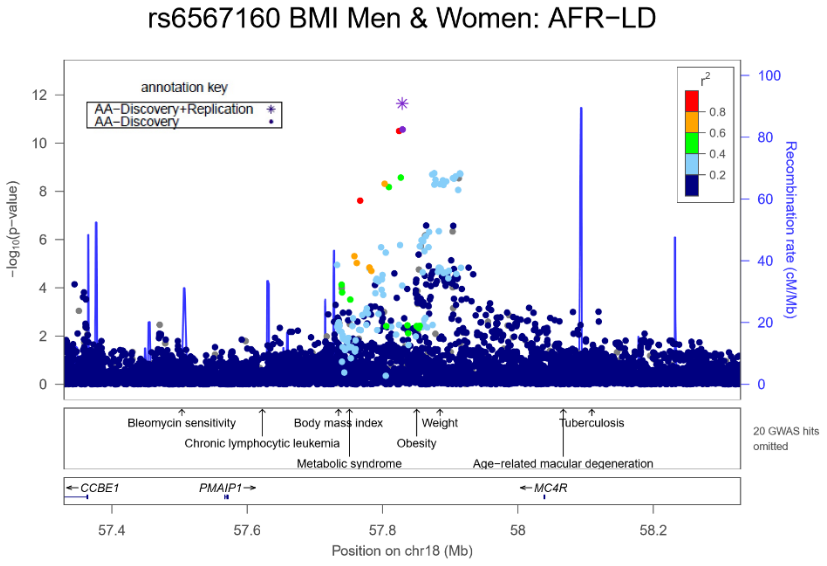
rs6567160 BMI Men & Women: AFR−LD
annotation key
AA−Discovery+Replication
AA−Discovery
−log10(p−value)
Recombination rate (cM/Mb)
Bleomycin sensitivity
Body mass index
Weight
Tuberculosis
Chronic lymphocytic leukemia
Obesity
Metabolic syndrome
Age−related macular degeneration
20 GWAS hits omitted
CCBE1
PMAIP1
MC4R
Position on chr18 (Mb)

(B)

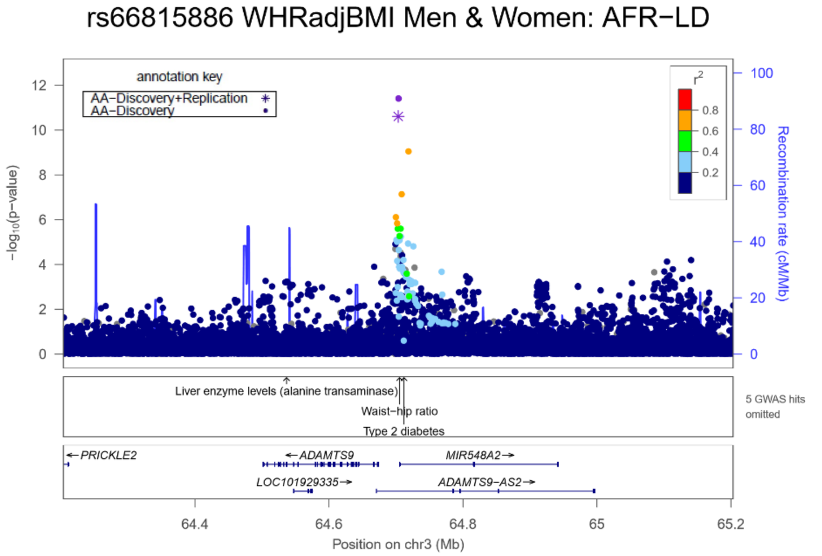
rs66815886 WHRadjBMI Men & Women: AFR−LD
annotation key
AA−Discovery+Replication
AA−Discovery
−log10(p−value)
Recombination rate (cM/Mb)
Liver enzyme levels (alanine transaminase)
Waist−hip ratio
Type 2 diabetes
5 GWAS hits omitted
PRICKLE2
ADAMTS9
MIR548A2
LOC101929335
ADAMTS9−AS2
Position on chr3 (Mb)
