# Supplementary material for: Discovery and fine-mapping of adiposity loci using high density imputation of genome-wide association studies in individuals of African ancestry: African Ancestry Anthropometry Genetics Consortium
Source: PLoS Genet. 2017 Apr 21;13(4):e1006719. doi: 10.1371/journal.pgen.1006719 (PMC5419579; doi:10.1371/journal.pgen.1006719)
Supplement: S11 Fig — Correlation of effect sizes for (A) BMI and (B) WHRadjBMI, and effect allele frequencies for (C) BMI and (D) WHRadjBMI in European and African ancestry studies in SNP transferability analyses. (PDF) [file pgen.1006719.s011.pdf]

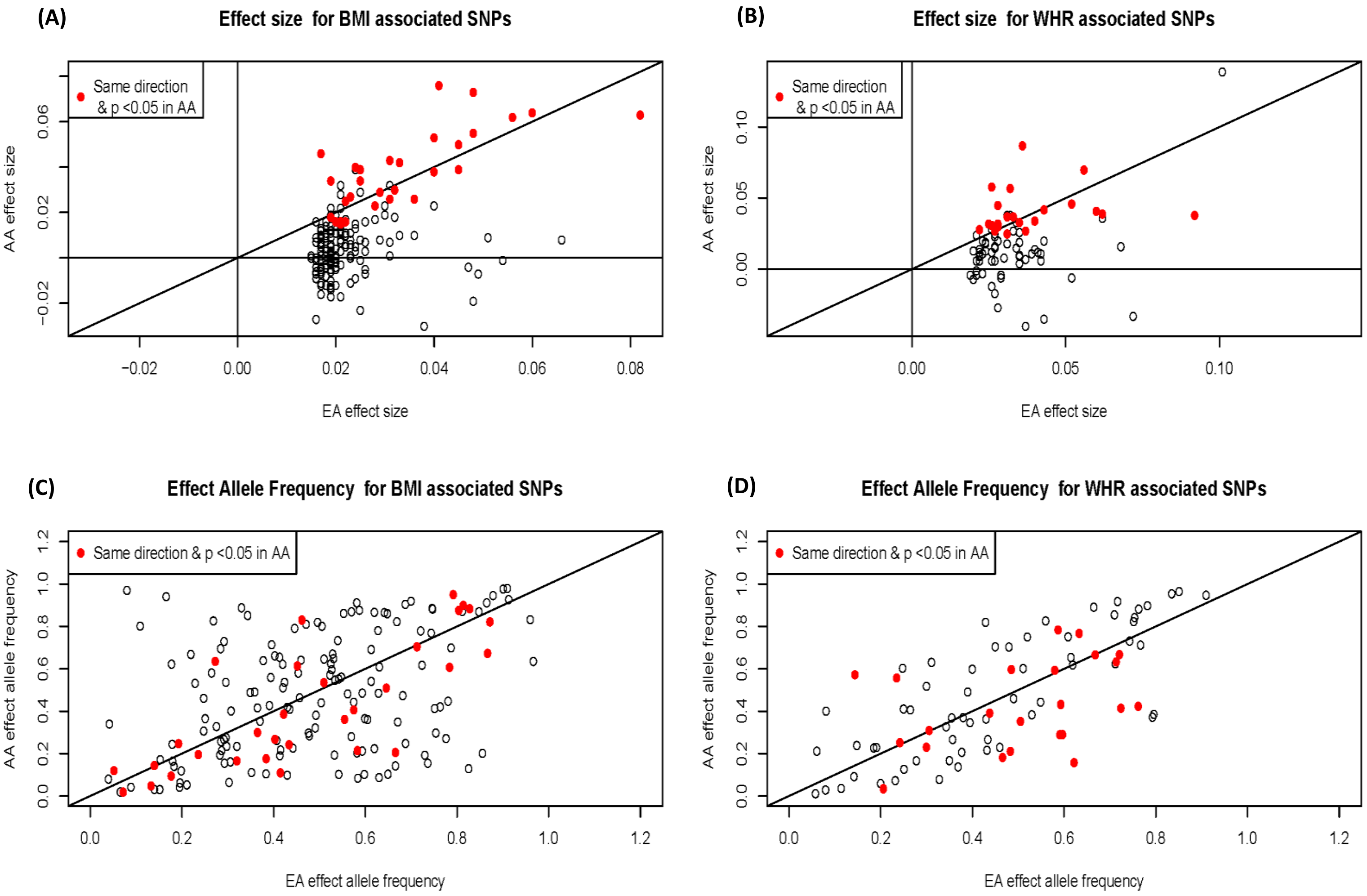
(A)
Effect size for BMI associated SNPs
Same direction
& p <0.05 in AA
AA effect size
EA effect size
(B)
Effect size for WHR associated SNPs
Same direction
& p <0.05 in AA
AA effect size
EA effect size
(C)
Effect Allele Frequency for BMI associated SNPs
Same direction & p <0.05 in AA
AA effect allele frequency
EA effect allele frequency
(D)
Effect Allele Frequency for WHR associated SNPs
Same direction & p <0.05 in AA
AA effect allele frequency
EA effect allele frequency
