# Supplementary material for: Discovery and fine-mapping of adiposity loci using high density imputation of genome-wide association studies in individuals of African ancestry: African Ancestry Anthropometry Genetics Consortium
Source: PLoS Genet. 2017 Apr 21;13(4):e1006719. doi: 10.1371/journal.pgen.1006719 (PMC5419579; doi:10.1371/journal.pgen.1006719)
Supplement: S1 Text — (DOCX) [file pgen.1006719.s026.docx]

**Supplementary Note**

**Description of Stage 1 Discovery Studies**

**Genome-wide association study of breast cancer in African Americans (AABC):** AABC consists of individuals from the nine studies described below [1]. All studies had weight and height information and three studies had information on waist and hip measurements and contributed to the GWAS. In these studies, weight and height information was based on self-report and waist and hip circumferences were measured.

*The Carolina Breast Cancer Study (CBCS):* The CBCS is a population-based case-control study conducted between 1993 and 2001 in 24 counties of central and eastern North Carolina [2]. Cases were identified by rapid case ascertainment system in cooperation with the North Carolina Central Cancer Registry and controls were selected from the North Carolina Division of Motor Vehicle and United States Health Care Financing Administration beneficiary lists. Participants’ ages ranged from 20 to 74 years. For stage 1, DNA samples were provided from 656 African American cases with invasive breast cancer and 608 African American controls. The study was approved by the institutional review board at the University of North Carolina.

*The Los Angeles component of The Women’s Contraceptive and Reproductive Experiences Study (CARE):* The NICHD Women's CARE Study is a large multi-center population-based case-control study that was designed to examine the effects of oral contraceptive use on invasive breast cancer risk among African American women and white women ages 35-64 years in five U.S. locations [3]. Cases in Los Angeles County were diagnosed from July 1, 1994 through April 30, 1998, and controls were sampled by random-digit dialing (RDD) from the same population and time period.

T*he Multiethnic Cohort Study (MEC):* The MEC is a prospective cohort study of 215,000 men and women in Hawaii and Los Angeles between the ages of 45 and 75 years at baseline (1993-1996) [4]. Through December, 31 2007, a nested breast cancer (BC) case-control study in the MEC included 556 African American cases (544 invasive and 12 in situ) and 1,003 African American controls. The study was approved by the institutional review board at the University of Southern California Institutional Review Board.

*The Nashville Breast Health Study (NBHS):* The NBHS is a population-based case-control study of breast cancer conducted in Tennessee [5]. The study was initiated in 2001 to recruit patients with invasive breast cancer or ductal carcinoma in situ, and controls, recruited through RDD between the ages of 25 and 75 years.

*The Northern California Breast Cancer Family Registry (NC-BCFR):* The NC-BCFR is a population based family study conducted in the Greater San Francisco Bay Area, and is one of 6 sites collaborating in the Breast Cancer Family Registry (BCFR), an international consortium funded by NCI [6]. African American breast cancer cases in NC-BCFR were diagnosed after January 1, 1995 and between the ages of 18 and 64 years; population controls were identified through RDD.

*The Prostate, Lung, Colorectal, and Ovarian Cancer Screening Trial (PLCO) Cohort:* PLCO, coordinated by the U.S. National Cancer Institute (NCI) in 10 U.S. centers, enrolled during 1993 – 2001 approximately 155,000 men and women, aged 55-74 years, in a randomized, two-arm trial to evaluate the efficacy of screening for these four cancers [7].

*The San Francisco Bay Area Breast Cancer Study (SFBCS):* The SFBCS is a population-based case control study of invasive breast cancer in Hispanic, African American and non-Hispanic White women conducted between 1995 and 2003 in the San Francisco Bay Area [8]. African American cases, ages 35-79 years, were diagnosed between April 1, 1995 and April 30, 1999, with controls identified through RDD.

*Wake Forest University Breast Cancer Study (WFBC):* African American breast cancer cases and controls in WFBC were recruited at Wake Forest University Health Sciences from November 1998 through December 2008 [9]. Controls were recruited from the patient population receiving routine mammography at the Breast Screening and Diagnostic Center. Age range of participants was 30-86 years.

*The Women’s Circle of Health Study (WCHS):* The WCHS is a case-control study of breast cancer in the New York City boroughs (Manhattan, the Bronx, Brooklyn and Queens) and in seven counties in New Jersey (Bergen, Essex, Hudson, Mercer, Middlesex, Passaic, and Union) [10]. Eligible cases included women with invasive breast cancer between 20 and 74 years of age; controls were identified through RDD. The study was approved by the institutional review board at Roswell Park Cancer Institute.

**Genome-wide association study of prostate cancer in African Americans (AAPC):** AAPC includes individuals from the case-control studies of prostate cancer in men of African ancestry described below. In all AAPC studies information on weight and height was based on self-report (except PCBP). Two of these studies had information on waist and hip measurements and contributed to the GWAS. Waist and hip circumferences were measured in both of these studies (MEC and MDA).

*The Cancer Prevention Study II Nutrition Cohort (CPS-II):* The CPS-II Nutrition Cohort includes over 86,000 men and 97,000 women from 21 US states who completed a mailed questionnaire in 1992 (aged 40-92 years at baseline) [11]. Starting in 1997, follow-up questionnaires were sent to surviving cohort members every other year to update exposure information and to ascertain occurrence of new cases of cancer; a >90% response rate has been achieved for each follow-up questionnaire. From 1998-2001, blood samples were collected in a subgroup of 39,376 cohort members. To further supplement the DNA resources, during 2000-2001, buccal cell samples were collected by mail from an additional 70,000 cohort members. Incident cancers are verified through medical records, or through state cancer registries or death certificates when the medical record cannot be obtained.

*Case-Control Study of Prostate Cancer among African Americans in Washington, DC (DCPC):* Unrelated men self-described as African American were recruited for several case-control studies on genetic risk factors for prostate cancer between the years 2001 and 2005 from the Division of Urology at Howard University Hospital (HUH) in Washington, DC. Control subjects unrelated to the cases and matched for age (± 5 years) were also ascertained from the prostate cancer screening population of the Division of Urology at HUH [12].

The Gene-Environment Interaction in Prostate Cancer Study (GECAP): The Henry Ford Health System (HFHS) recruited cases diagnosed with adenocarcinoma of the prostate of Caucasian or African-American race, less than 75 years of age, and living in the metropolitan Detroit tri-county area [13]. Controls were randomly selected from the same HFHS population base from which cases were drawn. The control sample was frequency matched at a ratio of 3 enrolled cases to 1 control based on race and five-year age stratum. In total, 637 cases and 244 controls were enrolled between January 2002 and December 2004.

*King County (Washington) Prostate Cancer Studies (KCPCS):* The study population consists of participants from one of two population-based case-control studies among residents of King County, Washington [14,15]. Incident Caucasian and African American cases with histologically confirmed prostate cancer were ascertained from the Seattle-Puget Sound SEER cancer registry during two time periods, 1993-1996 and 2002-2005. Age-matched (5-year age groups) controls were men without a self-reported history of being diagnosed with prostate cancer and were identified using one-step random digit telephone dialing. Controls were ascertained during the same time periods as the cases. Detailed in-person interviews collected self-reported data on weight one year prior to reference date (date of diagnosis for cases and a randomly assigned date for controls that approximated the distribution of diagnosis dates of cases) and maximum adult height.

*The Los Angeles Study of Aggressive Prostate Cancer (LAAPC):* The LAAPC is a population-based casecontrol study of aggressive prostate among African Americans in Los Angeles County [16]. Cases were identified through the Los Angeles County Cancer Surveillance Program rapid case ascertainment system and eligible cases included African American men diagnosed with a first primary prostate cancer between January 1, 1999 and December 31, 2003. Eligible cases also had either tumor extension outside the prostate, metastatic prostate cancer in sites other than prostate, or needle biopsy of the prostate with Gleason grade 8 or higher, or Gleason grade 7 and tumor in more than 2/3 of the biopsy cores. Controls were identified by a neighborhood walk algorithm and were men never diagnosed with prostate cancer, and were frequency matched to cases on age (±5 years).

*The Multiethnic Cohort (MEC):* The MEC is a prospective cohort study of 215,000 men and women in

Hawaii and Los Angeles1 between the ages of 45 and 75 years at baseline (1993-1996).

*Prostate Cancer Genetics Study (CaP Genes):* The African-American component of this study population comprised 160 men: 75 cases diagnosed with more aggressive prostate cancer and 85 age-matched controls [17]. All subjects were recruited and frequency-matched on the major medical institutions in Cleveland, Ohio (i.e., the Cleveland Clinic, University Hospitals of Cleveland, and their affiliates) between 2001 and 2004. The cases were newly diagnosed with histologically confirmed disease: Gleason score 7; tumor stage T2c; or a prostate-specific antigen level >10 ng/ml at diagnosis. Controls were men without a prostate cancer diagnosis who underwent standard annual medical examinations at the collaborating medical institutions.

*Prostate Cancer in a Black Population (PCBP):* The PCBP is a population-based case-control study of prostate cancer conducted in Barbados, West Indies. The study (2002-2011) included all incident, histologically-confirmed cases of prostate cancer ascertained from the Pathology Department of the Queen Elizabeth Hospital, Bridgetown, the only institution on the island where specimens are evaluated. Controls were randomly selected from a national database and frequency matched (by 5-year age groups) to the cases. Weight was measured in pounds using a beam balance scale and height was measured in centimeters using a metric rule attached to a wall and a right-angled wood block.

*Prostate Cancer Case-Control Studies at MD Anderson (MDA):* Participants in this study were identified from epidemiological prostate cancer studies conducted at the University of Texas M.D. Anderson Cancer Center in the Houston Metropolitan area since 1996. Cases were accrued from six institutions in the Houston Medical Center and were not restricted with respect to Gleason score, stage or PSA. Controls were identified via random-digit-dialing or among hospital visitors and they were frequency matched to cases on age and race. Lifestyle, demographic, and family history data were collected using a standardized questionnaire [18]. The study was approved by the institutional review board at the University of Texas MD Anderson Cancer Center.

*Selenium and Vitamin E Cancer Prevention Trial (SELECT):* SELECT is a phase III, placebo-controlled trial that tested whether selenium and vitamin E alone or in combination, might reduce the risk of developing prostate cancer[19]. A total of 35,534 men 55 and older (50 years and older for African Americans) without a history of prostate cancer were enrolled between 2001 and 2004. About 12% of the SELECT participants are African American. A case-cohort study has been established in SELECT.

*The Southern Community Cohort Study (SCCS):* The SCCS is a prospective cohort of African Americans

and non-African Americans which during 2002-2009 enrolled approximately 86,000 residents aged 40-79

years across 12 southern states [20]. Recruitment occurred mainly at community health centers, institutions providing basic health services primarily to the medically uninsured, so that the cohort includes many adults of lower income and educational status. Each study participant completed a detailed baseline questionnaire, and nearly 90% provided a biologic specimen (approximately 45% a blood sample and 45% buccal cells). Follow-up of the cohort is conducted by linkage to national mortality registers and to state cancer registries. Height and weight self-reported for the majority of study participants. For those who had clinic appointments on the day of interview, weight and height were abstracted from their medical record for that day and were found to be highly correlated (>.95) with self-reported values. For approximately 10% of those enrolled, the study interviewers measured height, weight and waist and hip circumference.

**Atherosclerosis Risk in Communities (ARIC) Study:** The ARIC study is a prospective population-based study of atherosclerosis and cardiovascular diseases in 15,792 men and women, including 11,478 non-Hispanic whites and 4,314 African Americans, drawn from 4 U.S. communities (suburban Minneapolis, Minnesota; Washington County, Maryland; Forsyth County, North Carolina, and Jackson, Mississippi) [21]. In the first three communities, the sample reflects the demographic composition of the community. In Jackson, only black residents were enrolled. Because of the design, only self-reported African-American participants are included in this analysis. Participants were between age 45 and 64 years at their baseline examination in 1987-1989 when blood was drawn for DNA extraction and participants consented to genetic testing. After taking into account availability of adequate amounts of high quality DNA, appropriate informed consent and genotyping quality control and assurance procedures, genotype data were available on 2,848 African-American individuals. Weight was measured to the nearest pound using a balance scale. Height was measured to the nearest centimeter using a wall-mounted ruler. Both weight and height were measured without shoes. Waist circumference (WC) was measured at the level of the umbilicus at the end of an exhalation to the nearest centimeter; hip girth was measured at the level of maximal protrusion of the gluteal muscles (hips). Both waist and hip circumferences were measured to the nearest centimeter, rounding down. Waist-hip ratio (WHR) was computed as waist circumference (cm) divided by hip circumference (cm).

**Mount Sinai Bio*Me* BioBank:** The Institute for Personalized Medicine (IPM) Bio*Me* Biobank Project is a consented, EMR-linked medical care setting biorepository of the Mount Sinai Medical Center (MSMC) drawing from a population of over 70,000 inpatients and 800,000 outpatient visits annually. MSMC serves diverse local communities of upper Manhattan, including Central Harlem (86% African American), East Harlem (88% Hispanic Latino), and Upper East Side (88% Caucasian/white) with broad health disparities. IPM Biobank populations include 28% African American, 38% Hispanic Latino predominantly of Caribbean origin, 23% Caucasian/White. IPM Biobank disease burden is reflective of health disparities with broad public health impact. Biobank operations are fully integrated in clinical care processes, including direct recruitment from clinical sites waiting areas and phlebotomy stations by dedicated Biobank recruiters independent of clinical care providers, prior to or following a clinician standard of care visit. Recruitment currently occurs at a broad spectrum of over 30 clinical care sites. Height and Weight data are from questionnaires submitted at the time of Biobank enrollment. Waist and hip circumference were not assessed.

**Coronary Artery Risk Development in young Adults (CARDIA):** The CARDIA study is a prospective, multi-center investigation of the natural history and etiology of cardiovascular disease in African Americans and whites 18-30 years of age at the time of initial examination. The initial examination included 5,115 participants selectively recruited to represent proportionate racial, gender, age, and education groups from four communities: Birmingham, AL; Chicago, IL; Minneapolis, MN; and Oakland, CA. Participants from the Birmingham, Chicago, and Minneapolis centers were recruited from the total community or from selected census tracts. Participants from the Oakland center were randomly recruited from the Kaiser-Permanente health plan membership. Details of the study design have been published [22]. From the time of initiation of the study in 1985-1986, five follow-up examinations have been conducted at years 2, 5, 7, 10, 15, and 20. DNA extraction for genetic studies was performed at the Y10 examination. After taking into account availability of adequate amounts of high quality DNA, appropriate informed consent and genotyping quality control and assurance procedures, genotype data were available on 952 African-American individuals. Each participant’s age, race, and sex were self-reported during the recruitment phase and verified during the baseline clinic visit. Weight was measured using a *Detecto* Scale (Model #68965) with additional weights (#68967) to allow weighing up to 450 pounds. Height was measured using an anthropometric ruler in centimeters or stadiometer. Measurements were made while participants were wearing minimal clothing (short sleeve shirt, shorts, and socks) and without shoes. Waist and hip girth were measured using a Gulick II Plus 300 cm anthropometric tape to the nearest 1/2 cm. Waist girth was obtained with the tape horizontally at a level laterally that is midway between the iliac crest and the lowest lateral portion of the rib cage and anteriorly midway between the xiphoid process of the sternum and the umbilicus; hip girth was obtained at the level of the symphysis pubis anteriorly and posteriorly at the level of the maximal protrusion of the gluteal muscles.

**Cleveland Family Study (CFS):** The Cleveland Family Study (CFS) is a family-based, longitudinal study designed to characterize the genetic and non-genetic risk factors for sleep apnea [23]. In total, 2534 individuals (46% African American) from 352 families were studied on up to 4 occasions over a period of 16 years (1990-2006). The initial aim of the study was to quantify the familial aggregation of sleep apnea. Over time, the aims were expanded to characterize the natural history of sleep apnea, sleep apnea outcomes, and to identify the genetic basis for sleep apnea. With subsequent exams, the cohort was expanded to include increased minority representation and additional family members. The total sample included index probands (N=275) who were recruited from 3 area hospital sleep centers if they had a confirmed diagnosis of sleep apnea and at least 2 first-degree relatives available to be studied. In the first 5 years of the study, neighborhood control probands (N=87) with at least 2 living relatives available for study were selected at random from a list provided by the index family. All available first-degree relatives and spouses of the case and control probands were recruited. Second-degree relatives, including half-sibs, aunts, uncles and grandparents, were also included if they lived near the first degree relatives (cases or controls), or if the family had been found to have two or more relatives with sleep apnea. The sample, which is enriched for individuals with sleep apnea, also contains a high prevalence of individuals with sleep apnea-related traits, including: obesity, impaired glucose tolerance, and hypertension. Data that were used for this analyses were for individuals in whom DNA had been collected (i.e., over the last two exam cycles (N=1447). Height was measured using a rigid stadiometer. Weight was measured with a calibrated digital scale. Waist circumference was measured at the narrowest point of the torso between the rib cage and the iliac crest to the nearest 0.1 cm. Hip circumference was measured at the level of maximal protrusion of the gluteal muscles to the nearest 0.1 cm. All measurements were made in duplicate and averaged. Data from the last available exam (N=696) was used for all analyses.

**The Cardiovascular Health Study (CHS):** The CHS is a prospective population-based cohort study of risk factors for CHD and stroke in adults 65 years and older. In June 1990, four Field Centers completed the recruitment of 5201 participants [24]. Between November 1992 and June 1993, an additional 687 African Americans were recruited using similar methods. The Field Centers are located in Forsyth County, NC; Sacramento County, CA; Washington County, MD; and Pittsburgh, PA. The baseline examinations consisted of a home interview and a clinic examination. Weight was measured to the nearest 0.1 Lb with the participant wearing light clothes. Height was measured to the nearest 0.1 cm. Body Mass Index was computed as weight (in kg) divided by height squared (in meters). Both waist (WC) and hip circumferences were measured to the nearest centimeter. Waist-hip ratio (WHR) was computed as waist circumference (cm) divided by hip circumference (cm). Data from the baseline visit was used for all analyses.

**The Family Heart Study (FamHS):** The Family Heart Study began in 1992, and subjects were recruited for an extensive clinical examination during the years 1994-96 [25]. A second visit, approximately 8 years later, was conducted, in which a sample of African-American families (N=624) was recruited at the University of Alabama in Birmingham field center. The primary purpose of the visit was to obtain two sequential cardiac multidetector CT (MDCT) exams for individual coronary arteries (i.e., left main, left anterior descending, diagonals, circumflex and right coronary arteries) and the aorta to estimate subclinical atherosclerosis burden as calcification. Measurements of the most important CHD risk factors in the lipid, glucose metabolism, blood pressure, and anthropometry domains also were assessed, along with medical history and medication use. Anthropometric measurements were performed with the participants wearing a scrub suit or examination gown, non-constricting underwear and no shoes. Participants were given the opportunity to empty their bladder before taking measurements. Weight was measured to the nearest pound on a calibrated balance scale. Height was measured without shoes to the nearest centimeter with a stadiometer. Body Mass Index (BMI) was computed as weight (in kg) divided by height^­­­^ squared (in meters). Waist circumference (WC) was measured at the level of the umbilicus at the end of an exhalation to the nearest centimeter; hip girth was measured at the level of maximal protrusion of the gluteal muscles (hips). Both waist and hip circumferences were measured to the nearest centimeter, rounding down. Waist-hip ratio (WHR) was computed as waist circumference (cm) divided by hip circumference (cm).

**Genetic Study of Atherosclerosis Risk (GeneSTAR):** GeneSTAR is an ongoing prospective study begun in 1983 designed to determine environmental, phenotypic, and genetic causes of premature cardiovascular disease[26,27]. Participants came from families identified from 1983-2006 from probands with a premature coronary disease event prior to 60 years of age who were identified at the time of hospitalization in any of 10 Baltimore area hospitals. Their apparently healthy 30-59 year old siblings without known CAD were recruited and underwent phenotypic measurement and characterization between 1983 and 2006. From 2003-2006, adult offspring over 21 years of age of all participating siblings and probands and the coparent of the offspring were recruited and underwent phenotypic measurement and characterization. For this study, only African American participants were included. Weight was measured in pounds using a clinical balance scale with participants wearing light, indoor clothing. Height was measured in inches using a stadiometer. Waist circumference (WC) was measured in inches in the standing position at the level of the iliac crest, and hip circumference was measured in inches at the widest level. The average of two measurements for the waist and hips was used; a third measurement was taken if the first two measurements varied by half an inch or more. Waist to hip ratio (WHR) was calculated as waist(cm)/hip(cm). Data from the first visit was used for all analyses.

**Health, Aging, and Body Composition (Health ABC) Study:** The Health ABC study is a prospective cohort study investigating the associations between body composition, weight-related health conditions, and incident functional limitation in older adults.  Health ABC enrolled well-functioning, community-dwelling black (N=1281) and white (N=1794) men and women aged 70-79 years between April 1997 and June 1998.  Participants were recruited from a random sample of white and all black Medicare eligible residents in the Pittsburgh, PA, and Memphis, TN, metropolitan areas.  Participants have undergone annual exams and semi-annual phone interviews.  Height and weight were measured with participants wearing light clothing and no shoes. BMI was computed as weight in kilograms divided by height in meters squared. Data from the baseline visit was used for all analyses. No hip measurement was collected in Health ABC.

**The Healthy Aging in Neighborhoods of Diversity across the Life Span study (HANDLS) study:**  The HANDLS study is an interdisciplinary, community-based, prospective longitudinal epidemiologic study examining the influences of race and socioeconomic status (SES) on the development of age-related health disparities among socioeconomically diverse African Americans and whites in Baltimore [28]. A total of 3,722 participants were recruited from Baltimore, MD with mean age 47.7 (range 30-64) years. We used a Health O Meter Digital Lithium Scale (Model #HDL 976) to measure weight in kg and a Novel Products Inc. Height Meter (Model #DES 290237) to measure height in cm. Participants stood erect on the floor with their heels placed together and touching the wall behind them and their back against the vertical height meter, which was mounted securely on the wall. Technician recorded centimeters when the height meter level was parallel to the floor. Participants stood on the scale, which was placed flat on a level surface with nothing underneath.  The scale was zeroed each morning.  Readings were taken in kilograms. Waist circumference was measured at the umbilicus at maximal circumference from exhalation, hip circumference was measured at the maximum circumference of the gluteal muscles, both to the nearest centimeter.

**Jackson Heart Study (JHS):** The Jackson Heart Study (JHS) is a prospective population-based study to seek the causes of the high prevalence of common complex diseases among African Americans in the Jackson, Mississippi metropolitan area, including cardiovascular disease, type-2 diabetes, obesity, chronic kidney disease, and stroke [29]. During the baseline examination period (2000-2004) 5,301 self-identified African Americans were recruited from four sources, including (1) randomly sampled households from a commercial listing; (2) ARIC participants; (3) a structured volunteer sample that was designed to mirror the eligible population; and (4) a nested family cohort. Unrelated participants were between 35 and 84 years old, and members of the family cohort were ≥ 21 years old when consent for genetic testing was obtained and blood was drawn for DNA extraction. Based on DNA availability, appropriate informed consent, and genotyping results that met quality control procedures, genotype data were available for 3,030 individuals, including 885 who are also ARIC participants. In the current study, JHS participants who were also enrolled in the ARIC study were analyzed with the ARIC dataset – for this reason, the JHS dataset analyzed here had 2,132 individuals. Weight was measured using a calibrated balance scale (Detector model #437), wearing light clothing and no shoes. Height was measured without shoes using a vertical ruler. Two measures of waist (horizontally at the level of the umbilicus, in the standing position) and hip (at the level of the widest circumference over the greater trochanter) were averaged to determine baseline WC and hip circumference, respectively, for each participant. Both circumferences were measured at the end of a gentle expiration while the participant was standing.

**Maywood Cohort:**  The Maywood cohort consisted of African Americans from Maywood, IL, who were enrolled in studies of blood pressure at the Loyola University Chicago Stritch School of Medicine, Maywood, IL, USA [30]. Study protocols were reviewed and approved by the institutional review board at the Loyola University Chicago Stritch School of Medicine prior to all recruitment activities. Written informed consent was obtained from each participant. Phenotype measurements were performed using a standardized protocol [31,32]. Body weight was measured to the nearest 0.2 kg on calibrated electronic scales, while height was obtained using a stadiometer consisting of a steel tape attached to a straight wall and a wooden headboard. The headboard was positioned with the participant shoeless, feet and back against the wall, and head held in the Frankfort horizontal plane and measurement taken to the nearest 0.1 cm [33]. Body mass index was calculated as the ratio of weight in kilograms to the square of height in meters. Waist and hip circumference were not assessed. A total of 653 unrelated participants with GWAS data were included in the present study.

**Multi-Ethnic Study of Atherosclerosis (MESA):** The Multi-Ethnic Study of Atherosclerosis (MESA) is a study of the characteristics of subclinical cardiovascular disease (disease detected non-invasively before it has produced clinical signs and symptoms) and the risk factors that predict progression to clinically overt cardiovascular disease or progression of the subclinical disease[34]. MESA researchers study a diverse, population-based sample of 6,814 asymptomatic men and women aged 45-84 at baseline. Approximately 38% of the recruited participants are white, 28% African-American, 22% Hispanic, and 12% Asian, predominantly of Chinese descent. Participants provided informed consent for participation in DNA studies. For the current study, after taking into account availability of adequate amounts of high quality DNA, appropriate informed consent and genotyping quality control and assurance procedures, genotype data were available on 1,650 African-American individuals. Participants were recruited from six field centers across the United States: Wake Forest University, Columbia University, Johns Hopkins University, University of Minnesota, Northwestern University and University of California, Los Angeles. Each participant received an extensive physical exam to determine coronary calcification, ventricular mass and function, flow-mediated endothelial vasodilation, carotid intimal-medial wall thickness, lower extremity vascular insufficiency, arterial wave forms, electrocardiographic (ECG) measures, standard coronary risk factors, socio-demographic factors, lifestyle factors, and psychosocial factors. DNA is extracted and lymphocytes cryopreserved for study of candidate genes and genome-wide scanning. Participants are followed for identification and characterization of cardiovascular disease events, including acute myocardial infarction and other forms of coronary heart disease (CHD), stroke, and congestive heart failure; for cardiovascular disease interventions; and for mortality. In addition to the six Field Centers, MESA involves a Coordinating Center, a Central Laboratory, and Central Reading Centers for Computed Tomography (CT), Magnetic Resonance Imaging (MRI), Ultrasound, and Electrocardiography (ECG). Protocol development, staff training, and pilot testing were performed in the first 18 months of the study. The first examination took place over two years, from July 2000 - July 2002. It was followed by three additional examination periods: September 2002 – January 2004, February 2004 - July 2005 September 2005 - May 2007. Participants are contacted every 9 to 12 months throughout the study to assess clinical morbidity and mortality. NHLBI recently funded MESA II, which brought back all participants back for a fifth exam, starting in April 2010. Height and weight were measured with participants wearing light clothing and no shoes. Girths (waist at the umbilicus and hips at the maximal circumference of buttocks) were measured in the standing position to the nearest 0.1 cm using a Gulick II 150-cm anthropometric tape steel measuring tape with standard 4 oz. tension. No measurement was taken around clothing. Waist-hip ratio (WHR) was calculated from waist and hip girth measurements. Body mass index (BMI) was defined as weight in kilograms divided by height in meters squared.

**Nigerian Cohort:** The population sampling frame for the Nigerian cohort was provided by the International Collaborative Study on Hypertension in Blacks (ICSHIB) as described in detail elsewhere [35]. Participants of the Nigerian cohort were recruited from Igbo-Ora and Ibadan in southwest Nigeria as a long-term study on the environmental and genetic factors underlying hypertension [30]. The study protocol was reviewed and approved by the Institutional Review Board at Loyola University Medical Center, and the Joint Ethical Committee of the University of Ibadan/University College Hospital, Ibadan, Nigeria. All participants gave written informed consent administered in either English or Yoruba. Phenotype measurements were performed by trained research staff using a standardized protocol [31,32]. Body weight was measured to the nearest 0.2 kg on calibrated electronic scales, whereas height was obtained using a stadiometer consisting of a steel tape attached to a straight wall and a wooden headboard. The headboard was positioned with the participant shoeless, feet and back against the wall and head held in the Frankfort horizontal plane and measurement taken to the nearest 0.1 cm. Body mass index was calculated as the ratio of weight in kilograms to the square of height in meters. Waist and hip circumference were not assessed. A total of 1,056 unrelated adults with GWAS data were evaluated in the present analysis.

**Wake Forest University School of Medicine (WFSM) Study:**  The WFSM GWAS study is a case-control study for type 2 diabetes and nephropathy [36]. Non-diabetic subjects who reported no history of diabetes and renal disease were recruited from the community and internal medicine clinics at WFSM. Diabetic subjects with type 2 diabetes and end stage renal disease were recruited from dialysis facilities in the southeastern U.S. Patients with type 2 diabetes were diagnosed after the age of 25 and did not receive only insulin therapy since diagnosis. In addition, cases had to have at least one of the following three criteria for inclusion: a) type 2 diabetes diagnosed at least 5 years before initiating renal replacement therapy, b) background or greater diabetic retinopathy and/or c) ≥100 mg/dl proteinuria on urinalysis in the absence of other causes of nephropathy. All subjects were recruited in North Carolina, South Carolina, Georgia, Tennessee, or Virginia. Informed consent was obtained from all study participants. Recruitment and sample collection procedures were approved by the Institutional Review Boards at WFSM. Among 1994 participants in the GWAS study, 815 non-diabetic subjects and 899 subjects with type 2 diabetes and end stage renal disease who have information on weight and height were included in this study. Measurements are either by self-report or by stadiometer for height and calibrated scale for weight. Waist and hip circumference were not assessed.

**Women’s Health Initiative – SNP Health Association Resource (WHI-SHARe).** WHI is one of the largest (n=161,808) studies of women's health ever undertaken in the US [37]. There are two major components of WHI: (1) a Clinical Trial (CT) that enrolled and randomized 68,132 women ages 50 – 79 into at least one of three placebo-control clinical trials (hormone therapy, dietary modification, and calcium/vitamin D); and (2) an Observational Study (OS) that enrolled 93,676 women of the same age range into a parallel prospective cohort study. A diverse population including 26,045 (17%) women from minority groups were recruited from 1993-1998 at 40 clinical centers across the U.S. Of the CT and OS minority participants enrolled in WHI, 12,157 (including 8,515 self-identified African American and 3,642 self-identified Hispanic subjects) who had consented to genetic research were eligible for the WHI SHARe GWAS project. DNA was extracted by the Specimen Processing Laboratory at the Fred Hutchinson Cancer research Center (FHCRC) using specimens that were collected at the time of enrollment. Specimens were stored at -80°C. Blood samples for WBC analyses were collected at baseline. Height was measured at an in-person visit with a stadiometer and weight was measured at an in-person visit with a calibrated scale. Weight was taken after removing shoes, heavy clothing, and pocket contents on a calibrated digital scale and recorded to the nearest one-tenth kilogram. Height was taken using a wall-mounted stadiometer and recorded to nearest one-tenth centimeter. BMI was calculated from measured height and weight. Waist and hip circumference were collected using standard protocols. Study protocols and consent forms were approved by the Institutional Review Boards at all participating institutions.

**Description of Stage 2 Replication Studies**

**Health and Retirement Study (HRS):** The Health and Retirement Study (HRS) is a longitudinal survey of a representative sample of Americans over the age of 50. The current sample includes over 26,000 persons in 17,000 households. Respondents are interviewed every two years about income and wealth, health and use of health services, work and retirement, and family connections. DNA was extracted from buccal swabs or saliva collected during a face-to-face interview in the respondents' homes. These data represent respondents who provided DNA samples and signed consent forms in 2006 and 2008. Respondents were removed if they were less than 18 years of age or had missing genotype or phenotype data.

**Howard University Family Study (HUFS):** The HUFS is a population-based study of African American families enrolled from the Washington, D.C. metropolitan area [38]. In the first phase of recruitment, a randomly ascertained cohort of 350 African American families with members in multiple generations from the Washington, D.C. metropolitan area were enrolled and examined. Families were not ascertained based on any phenotype. In a second phase of recruitment, additional unrelated individuals from the same geographic area were enrolled. The total number of recruited individuals was 2,028, of which 1,976 remained after data cleaning. Weight was measured on an electronic scale to the nearest 0.1 kg with the participant wearing light clothes. Height was measured with a stadiometer to the nearest 0.1 cm with participants in bare feet. Waist circumference (WC) was measured at the level of the umbilicus at the end of an exhalation. Hip circumference was measured at the level of maximal protrusion of the gluteal muscles (hips). Both waist and hip circumferences were measured to the nearest centimeter. Waist-hip ratio (WHR) was computed as waist circumference (cm) divided by hip circumference (cm). Ethical approval was obtained from the Howard University Institutional Review Board and written informed consent was obtained from each participant.

**Hypertensive Genetic Epidemiology Network (HyperGEN):** HyperGEN is part of the Family Blood Pressure Program funded by the National Heart Lung and Blood Institute and was designed to study the genetics of hypertension and related conditions [39] . Participants were recruited from multiply-affected hypertensive sibships ascertained through population-based cohorts or from the community-at-large. The study was later extended to include siblings and offspring of the original sibpair. Probands were identified by the onset of hypertension before age 60 and the presence of at least one additional hypertensive sibling who was willing to participate. Participants with type 1 diabetes or advanced renal disease were excluded from HyperGEN. Recruitment, clinical measurement, and DNA isolation were completed in 2003. Two of four centers (AL, NC) recruited 1,277 African Americans, while three centers (NC, MN, and UT) recruited Caucasians. The study was approved by the University of Alabama’s Internal Review Board for Human Use, the Washington University Human Research Production Office, the University of North Carolina’s Office of Human Research Ethics and the Medical College of Wisconsin’s Office of Human Research Protection Program. All subjects provided written informed consent. Body mass index was computed as weight (in kg) divided by height^­­­^ squared (in meters). Waist circumference (WC) and hip girth were measured to the nearest centimeter, rounding down. Waist-hip ratio (WHR) was computed as waist circumference (cm) divided by hip circumference (cm).

**A Genome-Wide Association Study of Breast Cancer in the African Diaspora (ROOT):** The following studies are included in the ROOT consortium.

*The Nigerian Breast Cancer Study (NBCS):* The NBCS is an ongoing case-control study of breast cancer in Ibadan, Nigeria initiated in 1998 [40,41]. Breast cancer cases were 20 years or older, ascertained at the University College Hospital, Ibadan, which is the oldest tertiary hospital in Nigerian with a catchment population of approximate three million. Controls were recruited from a randomly selected community in one of the communities adjoining the hospital. The majority of the study subjects were Yoruba and Yoruba is one of the populations selected by the International HapMap Project to represent African continent.

*The Barbados National Cancer Study (BNCS):* BNCS is a population-based case-control study designed to evaluate risk factors for incident breast and prostate cancer in the predominantly African population of Barbados, West Indies [42]. Cases were identified through the only pathology department on the island, located at the Queen Elizabeth Hospital, and represented all histologically confirmed incident cases of breast cancers between July 2002 and March 2006. Controls were selected from a national database provided by the Barbados Statistical Services Department, and were frequency matched to breast cancer cases at a 2:1 ratio and by 5-year age groups.

*The Racial Variability in Genotypic Determinants of Breast Cancer Risk Study (RVGBC)*: RVGBC is a hospital-based genetic epidemiologic study conducted in Philadelphia and Detroit metropolitan areas from 1999 to 2003. Breast cancer cases were identified in the University of Pennsylvania Health System and Karmanos Cancer Institute. Local advertisement was also put to recruit breast cancer cases living in the Philadelphia and Detroit area. Controls were recruited in the same fashion as cases in these institutions except that they do not have breast cancer. Patients with breast cancer had to be diagnosed within 18 months of recruitment and have invasive ductal cancer. The study was designed to over-represent women diagnosed under age 40.

*The Baltimore Breast Cancer Study (BBCS):* BBCS is a case control study of breast cancer designed to identify and characterize markers of disease aggressiveness and poor outcome. Incident breast cancer cases and controls were recruited between February of 1993 and August of 2003 in six hospitals in the greater Baltimore area, including the University of Maryland Medical Center, the Baltimore Veterans Affairs Medical Center, Union Memorial Hospital, Mercy Medical Center, and the Sinai Hospital. Controls were frequency-matched to cases by race and age.

*The Chicago Cancer Prone Study (CCPS):* CCPS is an ongoing hospital-based case-control study designed to investigate the genetics of young-onset breast cancer. Cases with histologically confirmed breast cancer were enrolled through the Cancer Risk Clinic at the University of Chicago. Young-onset cases and African Americans were oversampled. Controls were gender- and age-matched with cases and enrolled from patients who visited the same hospital and were willing to donate blood for genetic studies.

*The Southern Community Cohort (SCCS):* The SCCS is a prospective cohort study initiated in 2002 focusing on investigating racial disparities in cancer risk [20]. A nested case-control study of breast cancer is included in this project. Cases were women who were diagnosed with breast cancer after the entered the cohort. Control subjects were selected randomly from those who were cancer-free at the time of the study and frequency-matched to case patients in a 1 to 2 ratio on age at enrollment , recruitment method, and sample type (blood/buccal cell).

**The Study of Asthma Phenotypes and Pharmacogenomic Interactions by Race-Ethnicity (SAPPHIRE):** SAPPHIRE is an ongoing prospective cohort study to determine the genetic determinants of inhaled corticosteroid response and other asthma-related phenotypes [43]. Participants (n=728) were age 12-56 years at the time of enrollment, had at least one physician diagnosis of asthma, and had no prior diagnosis (in the electronic medical record or by patient report) of chronic obstructive pulmonary disease (COPD) or congestive heart failure (CHF). Controls (n=284) were recruited from the same geographic area and were also age 12-56 years at the time of enrollment, but these individuals had no prior diagnosis of asthma, COPD, or CHF. All participants received care from a single, large health system covering southeast Michigan and metropolitan Detroit. At enrollment, all participants underwent an evaluation which included a staff-administered survey, lung function testing, and measurement of vital signs, height, and weight. Waist and hip circumference were not assessed. Genomic DNA was isolated from a blood specimen obtained at the initial visit, and genotyping was performed using the Affymetrix Axiom Genome-Wide Population-Optimized Human Array.

**Sea Islands Genetics Network - Reasons for Geographic and Racial Differences in Stroke** (SIGNET-REGARDS): REGARDS is an observational cohort of 30,239 AA and white men and women enrolled in their homes after a telephone interview in 2003-7 [44]. Participants were a national sample oversampled from the southeastern stroke belt (56%) and were 58% female and 42% black by design. Participants were followed every 6 months by telephone to ascertain health outcomes, with validation of stroke, coronary heart disease, death and other ancillary study endpoints. For SIGNET, we selected all AA REGARDS type 2 diabetes (T2D) cases recruited from SC, GA, NC, and AL, and an equivalent number of race, sex, and age-strata matched diabetes-free controls. We also included all participants not already included that were current residents of the 15-county “Low Country” region of SC and GA (SC counties Beaufort, Berkeley, Charleston, Colleton, Dorchester, Georgetown, Hampton, Horry, Jasper; GA counties Bryan, Camden, Chatham, Glynn, Liberty, McIntosh). The subset of REGARDS participants genotyped under SIGNET are referred to as SIGNET-REGARDS. GWAS genotyping was completed among 2398 SIGNET-REGARDS AA participants, including 1149 with diabetes and 1249 without diabetes. All samples in this project (n= 2348) were genotyped using the Affymetrix Genome-Wide Human SNP Array 6.0. Anthropometric measurements were made with participants wearing light-weight, non-constricting underwear and no shoes. Weight was measured to the nearest pound, and height without shoes to the nearest centimeter. Body Mass Index (BMI) was computed as weight (in kg) divided by height^­­­^ squared (in meters). Waist circumference (WC) was measured at the level of the umbilicus at the end of an exhalation to the nearest centimeter; hip girth was measured at the level of maximal protrusion of the gluteal muscles (hips). Both waist and hip circumferences were measured to the nearest centimeter, rounding down. Waist-hip ratio (WHR) was computed as waist circumference (cm) divided by hip circumference (cm).

**Description of Children Studies**

**Bone Mineral Density in Childhood Study (BMDCS):** BMDCS was a prospective, longitudinal study established to develop reference bone mineral density (BMD) and bone mineral content (BMC) growth charts in healthy U.S. children. The participants were recruited in 2002-2003 at five sites across the U.S. (Children’s Hospital of Los Angeles, Cincinnati Children’s Hospital Medical Center, Creighton University, Children’s Hospital of Philadelphia, and Columbia University Medical Center). The females were aged 6 to 15 years and the males were aged 6 to 16 years at enrollment, and they were followed-up annually for 6 years. In 2006-2007, 5- and 19-year-olds were additionally enrolled to extend the reference percentiles; these participants were follow-up annually for 2 years. To be enrolled the participants had to meet the following criteria: term birth (≥37 wk gestation), birth weight >2.3 kg, no evidence of precocious or delayed puberty, and height, weight or BMI within the 3rd to the 97th percentiles for age and sex. Participants were excluded at baseline if they had experienced more than two fractures by age 10 years or more than three fractures after age 10 years. Current or previous users of bone health medications or children who had a medical condition known to affect bone health were also excluded, as were children who had experienced extended bed rest. At the final study visit the participants were invited to provide a blood or saliva sample to allow for the extraction of DNA. We extracted DNA from blood or saliva and genome-wide SNP genotyped the DNA at CHOP’s Center for Applied Genomics, using the Illumina Infinium™ II OMNI Express plus Exome BeadChip technology (Illumina, San Diego)

**Children's Hospital of Philadelphia (CHOP):** All subjects were consecutively recruited from the Greater Philadelphia area from 2006 to 2012 at CHOP. Our study cohort consisted of children of African ancestry. All of these participants had their blood drawn in to an 8ml EDTA blood collection tube and were subsequently DNA extracted for genotyping. All subjects were biologically unrelated and were aged between 2 and 18 years old. This study was approved by the Institutional Review Board of the Children's Hospital of Philadelphia. Parental informed consent was given for each study participant for both the blood collection and subsequent genotyping. BMI was determined from height and weight measurements in the clinical record. We performed high throughput genome-wide SNP genotyping, using the Illumina Infinium™ II HumanHap550 BeadChip technology (Illumina, San Diego), at the Center for Applied Genomics at CHOP. We used 750ng of genomic DNA to genotype each sample, according to the manufacturer’s guidelines.

**References**

1. Chen F, Chen GK, Millikan RC, John EM, Ambrosone CB, et al. (2011) Fine-mapping of breast cancer susceptibility loci characterizes genetic risk in African Americans. Hum Mol Genet 20: 4491-4503.

2. Newman B, Moorman PG, Millikan R, Qaqish BF, Geradts J, et al. (1995) The Carolina Breast Cancer Study: integrating population-based epidemiology and molecular biology. Breast Cancer Res Treat 35: 51-60.

3. Marchbanks PA, McDonald JA, Wilson HG, Burnett NM, Daling JR, et al. (2002) The NICHD Women's Contraceptive and Reproductive Experiences Study: methods and operational results. Ann Epidemiol 12: 213-221.

4. Kolonel LN, Henderson BE, Hankin JH, Nomura AM, Wilkens LR, et al. (2000) A multiethnic cohort in Hawaii and Los Angeles: baseline characteristics. Am J Epidemiol 151: 346-357.

5. Zheng W, Cai Q, Signorello LB, Long J, Hargreaves MK, et al. (2009) Evaluation of 11 breast cancer susceptibility loci in African-American women. Cancer Epidemiol Biomarkers Prev 18: 2761-2764.

6. John EM, Miron A, Gong G, Phipps AI, Felberg A, et al. (2007) Prevalence of pathogenic BRCA1 mutation carriers in 5 US racial/ethnic groups. JAMA 298: 2869-2876.

7. Prorok PC, Andriole GL, Bresalier RS, Buys SS, Chia D, et al. (2000) Design of the Prostate, Lung, Colorectal and Ovarian (PLCO) Cancer Screening Trial. Control Clin Trials 21: 273S-309S.

8. John EM, Schwartz GG, Koo J, Wang W, Ingles SA (2007) Sun exposure, vitamin D receptor gene polymorphisms, and breast cancer risk in a multiethnic population. Am J Epidemiol 166: 1409-1419.

9. Smith TR, Levine EA, Freimanis RI, Akman SA, Allen GO, et al. (2008) Polygenic model of DNA repair genetic polymorphisms in human breast cancer risk. Carcinogenesis 29: 2132-2138.

10. Ambrosone CB, Ciupak GL, Bandera EV, Jandorf L, Bovbjerg DH, et al. (2009) Conducting Molecular Epidemiological Research in the Age of HIPAA: A Multi-Institutional Case-Control Study of Breast Cancer in African-American and European-American Women. J Oncol 2009: 871250.

11. Calle EE, Rodriguez C, Jacobs EJ, Almon ML, Chao A, et al. (2002) The American Cancer Society Cancer Prevention Study II Nutrition Cohort: rationale, study design, and baseline characteristics. Cancer 94: 2490-2501.

12. Robbins C, Torres JB, Hooker S, Bonilla C, Hernandez W, et al. (2007) Confirmation study of prostate cancer risk variants at 8q24 in African Americans identifies a novel risk locus. Genome Res 17: 1717-1722.

13. Rybicki BA, Neslund-Dudas C, Nock NL, Schultz LR, Eklund L, et al. (2006) Prostate cancer risk from occupational exposure to polycyclic aromatic hydrocarbons interacting with the GSTP1 Ile105Val polymorphism. Cancer Detect Prev 30: 412-422.

14. Agalliu I, Salinas CA, Hansten PD, Ostrander EA, Stanford JL (2008) Statin use and risk of prostate cancer: results from a population-based epidemiologic study. Am J Epidemiol 168: 250-260.

15. Stanford JL, Wicklund KG, McKnight B, Daling JR, Brawer MK (1999) Vasectomy and risk of prostate cancer. Cancer Epidemiol Biomarkers Prev 8: 881-886.

16. Ingles SA, Coetzee GA, Ross RK, Henderson BE, Kolonel LN, et al. (1998) Association of prostate cancer with vitamin D receptor haplotypes in African-Americans. Cancer Res 58: 1620-1623.

17. Liu X, Plummer SJ, Nock NL, Casey G, Witte JS (2006) Nonsteroidal antiinflammatory drugs and decreased risk of advanced prostate cancer: modification by lymphotoxin alpha. Am J Epidemiol 164: 984-989.

18. Strom SS, Gu Y, Zhang H, Troncoso P, Babaian RJ, et al. (2004) Androgen receptor polymorphisms and risk of biochemical failure among prostatectomy patients. Prostate 60: 343-351.

19. Lippman SM, Klein EA, Goodman PJ, Lucia MS, Thompson IM, et al. (2009) Effect of selenium and vitamin E on risk of prostate cancer and other cancers: the Selenium and Vitamin E Cancer Prevention Trial (SELECT). JAMA 301: 39-51.

20. Signorello LB, Hargreaves MK, Steinwandel MD, Zheng W, Cai Q, et al. (2005) Southern community cohort study: establishing a cohort to investigate health disparities. J Natl Med Assoc 97: 972-979.

21. The ARIC investigators (1989) The Atherosclerosis Risk in Communities (ARIC) Study: design and objectives. The ARIC investigators. Am J Epidemiol 129: 687-702.

22. Friedman GD, Cutter GR, Donahue RP, Hughes GH, Hulley SB, et al. (1988) CARDIA: study design, recruitment, and some characteristics of the examined subjects. J Clin Epidemiol 41: 1105-1116.

23. Tishler PV, Larkin EK, Schluchter MD, Redline S (2003) Incidence of sleep-disordered breathing in an urban adult population: the relative importance of risk factors in the development of sleep-disordered breathing. JAMA 289: 2230-2237.

24. Fried LP, Borhani NO, Enright P, Furberg CD, Gardin JM, et al. (1991) The Cardiovascular Health Study: design and rationale. Ann Epidemiol 1: 263-276.

25. Higgins M, Province M, Heiss G, Eckfeldt J, Ellison RC, et al. (1996) NHLBI Family Heart Study: objectives and design. Am J Epidemiol 143: 1219-1228.

26. Bordeaux BC, Qayyum R, Yanek LR, Vaidya D, Becker LC, et al. (2010) Effect of obesity on platelet reactivity and response to low-dose aspirin. Prev Cardiol 13: 56-62.

27. Mora S, Yanek LR, Moy TF, Fallin MD, Becker LC, et al. (2005) Interaction of body mass index and framingham risk score in predicting incident coronary disease in families. Circulation 111: 1871-1876.

28. Evans MK, Lepkowski JM, Powe NR, LaVeist T, Kuczmarski MF, et al. (2010) Healthy aging in neighborhoods of diversity across the life span (HANDLS): overcoming barriers to implementing a longitudinal, epidemiologic, urban study of health, race, and socioeconomic status. Ethn Dis 20: 267-275.

29. Taylor HA, Jr., Wilson JG, Jones DW, Sarpong DF, Srinivasan A, et al. (2005) Toward resolution of cardiovascular health disparities in African Americans: design and methods of the Jackson Heart Study. Ethn Dis 15: S6-4-17.

30. Kang SJ, Chiang CW, Palmer CD, Tayo BO, Lettre G, et al. (2010) Genome-wide association of anthropometric traits in African- and African-derived populations. Hum Mol Genet 19: 2725-2738.

31. Cooper R, Puras A, Tracy J, Kaufman J, Asuzu M, et al. (1997) Evaluation of an electronic blood pressure device for epidemiological studies. Blood Press Monit 2: 35-40.

32. Ataman SL, Cooper R, Rotimi C, McGee D, Osotimehin B, et al. (1996) Standardization of blood pressure measurement in an international comparative study. J Clin Epidemiol 49: 869-877.

33. Tayo BO, Harders R, Luke A, Zhu X, Cooper RS (2008) Latent common genetic components of obesity traits. Int J Obes (Lond) 32: 1799-1806.

34. Bild DE, Bluemke DA, Burke GL, Detrano R, Diez Roux AV, et al. (2002) Multi-Ethnic Study of Atherosclerosis: objectives and design. Am J Epidemiol 156: 871-881.

35. Cooper R, Rotimi C, Ataman S, McGee D, Osotimehin B, et al. (1997) The prevalence of hypertension in seven populations of west African origin. Am J Public Health 87: 160-168.

36. McDonough CW, Palmer ND, Hicks PJ, Roh BH, An SS, et al. (2011) A genome-wide association study for diabetic nephropathy genes in African Americans. Kidney Int 79: 563-572.

37. (1998) Design of the Women's Health Initiative clinical trial and observational study. The Women's Health Initiative Study Group. Control Clin Trials 19: 61-109.

38. Adeyemo A, Gerry N, Chen G, Herbert A, Doumatey A, et al. (2009) A genome-wide association study of hypertension and blood pressure in African Americans. PLoS Genet 5: e1000564.

39. Williams RR, Rao DC, Ellison RC, Arnett DK, Heiss G, et al. (2000) NHLBI family blood pressure program: methodology and recruitment in the HyperGEN network. Hypertension genetic epidemiology network. Ann Epidemiol 10: 389-400.

40. Huo D, Adebamowo CA, Ogundiran TO, Akang EE, Campbell O, et al. (2008) Parity and breastfeeding are protective against breast cancer in Nigerian women. Br J Cancer 98: 992-996.

41. Huo D, Kim HJ, Adebamowo CA, Ogundiran TO, Akang EE, et al. (2008) Genetic polymorphisms in uridine diphospho-glucuronosyltransferase 1A1 and breast cancer risk in Africans. Breast Cancer Res Treat 110: 367-376.

42. Nemesure B, Wu SY, Hambleton IR, Leske MC, Hennis AJ (2009) Risk factors for breast cancer in a black population--the Barbados National Cancer Study. Int J Cancer 124: 174-179.

43. Rumpel JA, Ahmedani BK, Peterson EL, Wells KE, Yang M, et al. (2012) Genetic ancestry and its association with asthma exacerbations among African American subjects with asthma. J Allergy Clin Immunol 130: 1302-1306.

44. Howard VJ, Cushman M, Pulley L, Gomez CR, Go RC, et al. (2005) The reasons for geographic and racial differences in stroke study: objectives and design. Neuroepidemiology 25: 135-143.
