# Supplementary material for: Discovery and fine-mapping of adiposity loci using high density imputation of genome-wide association studies in individuals of African ancestry: African Ancestry Anthropometry Genetics Consortium
Source: PLoS Genet. 2017 Apr 21;13(4):e1006719. doi: 10.1371/journal.pgen.1006719 (PMC5419579; doi:10.1371/journal.pgen.1006719)
Supplement: S2 Text — (DOCX) [file pgen.1006719.s027.docx]

**Members of the BMDCS Group**

Heidi Kalkwarf^1^, Joan Lappe^2^, Sharon Oberfield^3^, Vincent Gilsanz^4^, John Shepherd^5^, Andrea Kelly^6^

**1** UC Department of Pediatrics, Children's Hospital Medical Center Cincinnati, Cincinnati, OH, USA.

**2** Department of Medicine, Creighton University, Omaha, NE, USA.

**3** Division of Pediatric Endocrinology Diabetes and Metabolism, Columbia University Medical Center, New York, NY, USA.

**4** Department of Radiology, Children's Hospital Los Angeles, Los Angeles, CA, USA.

**5** Department of Radiology, University of California, San Francisco, San Francisco CA, USA.

**6** Division of Endocrinology and Diabetes, The Children's Hospital of Philadelphia, Philadelphia, PA, USA.
